# Supplementary material for: Long Covid symptoms and diagnosis in primary care: A cohort study using structured and unstructured data in The Health Improvement Network primary care database
Source: PLoS One. 2023 Sep 26;18(9):e0290583. doi: 10.1371/journal.pone.0290583 (PMC10521988; doi:10.1371/journal.pone.0290583)
Supplement: S1 Text — Supplementary methods and results. (PDF) [file pone.0290583.s001.pdf]

Supplementary Text

Supplementary Methods

Sample size calculation

We designed this study to have sufficient power for comparing the incidence of new symptoms or complications, or the proportion of patients experiencing a symptom within a particular time interval, between the exposed cohort and unexposed controls.

Assuming a sample size of 10,000 exposed patients and 10,000 unexposed patients, we calculated the power to detect differences in proportion between the two groups (e.g. the proportion of patients with mention of a symptom within a certain time interval). Using the normal approximation to the binomial distribution (R function ‘power.prop.test’ in the ‘stats’ package), using alpha = 0.05, we calculate the following:

Proportion among controls	Proportion among cases	Relative risk	Power
0.02	0.028	1.4	0.959
0.05	0.06	1.2	0.873
0.10	0.12	1.2	0.995

Free text extraction and data flows

We used data from The Health Improvement Network (THIN) primary care database (a Cegedim Database), including patients from England, Scotland, and Wales. The study period was 1 December 2019 to 31 December 2020, as this was the period for which unstructured text was available, but data prior to this period (such as historic diagnoses) was also used for baseline characterisation of patients.

Although THIN has the capability to extract free text information from primary care electronic health records, this can only be done with appropriate permissions (because free text may potentially contain information that may identify a person), and is resource intensive because of the large volume of data.

A data minimisation policy was used to minimise the risk of disclosure of patient identifiable information (see data flow diagram – Supplementary Figure S1). Free text was extracted only from patients that had already been selected based on queries on the structured data (i.e. Read codes in Supplementary Tables S1 and S2, or matched controls), and only for the time period 1 December 2019 to 31 December 2020. Free text was initially processed by an automated redaction algorithm which has been validated to remove at least 98% of direct identifiers such as name, date of birth etc. The text was then analysed by a rule-based named entity recognition and linking algorithm called the Freetext Matching Algorithm (FMA https://github.com/anoopshah/freetext-matching-algorithm) [1]. This algorithm was chosen because it has previously been validated on primary care free text. Although more machine learning algorithms are available (e.g. MedCAT https://github.com/CogStack/MedCAT), the lack of research access to UK primary care free text means that such algorithms have not yet been trained or validated on primary care data. Such algorithms should be used in future studies as their accuracy is likely to outperform the rule-based algorithm used in this study.

FMA is a rule-based named entity extraction and linking algorithm. The initial text preprocessing stage consists of conversion to lower case, stopword removal, identification of dates, durations and numbers using regular expressions, and spelling correction (attempting single letter insertion or substitution, except the first letter of the word). The algorithm then identifies context (e.g. negation, uncertainty and experiencer) by matching patterns against a manually curated set of expressions.

The next step is to match sequences of up to five words to Read terms. If the text phrase does not match a Read term exactly, parts of the phrase are substituted by alternative words and phrases using a manually curated synonym table. A similarity score between text phrases and candidate Read terms is used to find the best match out of a set of potential matches. More details about FMA are included in the program documentation on GitHub (https://github.com/anoopshah/freetext-matching-algorithm).

FMA was implemented within the THIN secure server to extract information about symptoms, hospitalisation and COVID-19 diagnosis to supplement the structured data. Algorithm outputs (consisting solely of structured data; extracted Read terms and dates) were transferred to the UCL data safe haven for researchers to analyse (see data flow diagram – Supplementary Figure S1).

Validity of information extracted from free text

Although the FMA has been developed and tested using primary care records, it has never been used on text from the time of a COVID-19 pandemic. We therefore carried out validation studies of random text samples from which certain items of information had been extracted. Samples for validation underwent an additional manual anonymisation process (dual independent checks by THIN staff) before researchers were permitted to view the text. We checked document-level precision only. We did not attempt to quantify recall as this would have required manual anonymisation of very large quantities of text (as the extracted items of information were sparse; we would have had to review the entire free text for a patient to verify that they did not have a symptom mentioned).

The COVID-19 concepts that could be extracted included the simple phrase ‘COVID-19’ in isolation, as well as longer phrases describing tests or complications of COVID (e.g. ‘COVID-19 pneumonia’). Natural language processing algorithms are prone to error if they do not correctly detect the context in which a concept is mentioned, such as whether it is a confirmed diagnosis for the patient, a topic of advice, a suspected diagnosis, or a hypothetical risk. For each COVID-19 related concept extracted from the text, we carried out an initial estimate of validity by calculating its association with the presence of a COVID-19 Read code. We expected that patients with a COVID-19 Read code (i.e. classified as exposed) would be much more likely to have COVID-19 concepts in the text as well. This was the case for most concepts, except the simple phrase ‘COVID-19’, which was recorded in half as many patients without a COVID-19 Read code as those with a COVID-19 Read code. We would expect this proportion to be much lower. This suggested that in many instances the algorithm was detecting generic text related to COVID-19 (e.g. ‘consultation by video because of COVID-19 pandemic’) rather than a diagnosis.

We carried out manual validation to calculate the precision of the following items extracted from free text. :

- Symptoms for suspected or confirmed COVID-19 cohorts (300, 10 with each of the 30 most common symptoms)
- Symptoms for unexposed cohort (300, 10 for each of the 30 most common symptoms)
- Simple ‘COVID-19’ concept with context detected as ‘confirmed’ (50, as described above we expected poor precision for this concept)
- Simple ‘COVID-19’ concept with context ‘suspected’ (250)
- Acute COVID-19 concept (e.g. ‘COVID-19 pneumonia’) with context ‘confirmed’ (250)
- Acute COVID-19 concept with context ‘suspected’ (250)
- Chronic COVID-19 concept - confirmed or suspected (250)

The sample size of 250 was chosen to provide 80% power to detect a difference between precision 0.86 and 0.94. Two researchers (ADS and JL) independently carried out the manual validation, and their findings were compared to calculate inter-rater reliability. After carrying out independent annotation, the annotators conferred and created a consensus manual annotation, against which the precision of the natural language processing algorithm was assessed.

Recording of symptoms

We used similar definitions of symptoms to a recent study by Subramanian et al. [2] using the Clinical Practice Research Datalink (CPRD) database, who studied 115 symptoms. However, given the smaller patient population in the current study, we combined some of the specific symptoms into more general symptoms (as in the cluster analysis of the CPRD study), resulting in a final list of 89 symptoms. For example, ‘Chest pain’ and ‘Pleuritic chest pain’ were combined. We used Read codelists created by Subramanian et al. (https://github.com/AnuSub/LongCOVID_Symptoms_CodeList) and sought to include most of the symptoms that may contribute to a Long Covid diagnosis according to World Health Organization (WHO) criteria [3].

We plotted the proportion of patients in each category with at least one symptom recorded, by 4-week period between 12 weeks before and 40 weeks after the index date. We calculated odds ratios for symptom recording compared to a reference period 8-12 weeks before the index date.

Calculation of propensity score

We sought to derive a propensity score for the probability of being in the COVID-19 exposed cohort. We used methods similar to those in the study by Subramanian et al. [2]; predictor variables for the propensity score included patient demographics and pre-existing medical conditions. However, as the complete set of medical conditions that increase the risk of COVID-19 infection is unknown, we used a data-driven approach to select the optimal set of disease variables to

include in the propensity score model. We used the SNOMED CT hierarchy to assist the model specification following our work on phenotype definition using SNOMED CT [4]. The SNOMED CT knowledge model includes representations of which disorders are subtypes of another, and therefore assists in grouping disorders where appropriate to create a parsimonious model. We used NHS Digital mappings to translate Read codes for disease diagnoses recorded in THIN to SNOMED CT concepts with the semantic type 'Disorder'. We then generated a set of binary variables per patient for the presence of each of the 1000 most common SNOMED CT disorder concepts at any time prior to the index date.

If a disorder was present for a patient, all ancestors of that disorder (broader, more general concepts) were also considered to be true for that patient. For example, if a patient had a Read code for 'Heart failure with reduced ejection fraction', they were also considered to have 'Disorder of cardiac function'. SNOMED CT based grouping of disease variables was used in combination with variable selection using LASSO, enabling the automatic selection of broader disease variables if multiple narrower disease variables did not provide additional predictive power.

We generated separate propensity scores for each of the comparisons investigated in this study:

- confirmed COVID-19 versus unexposed
- suspected COVID-19 versus unexposed
- viral or respiratory illness versus unexposed

We generated propensity scores using logistic regression with LASSO [5] to select appropriate predictors, allowing a maximum of 100 predictors in models, using 10-fold cross validation for parameter tuning. Potential predictors included age, sex, ethnicity, smoking, deprivation and body mass index (which were forced to be included in the models by setting a penalty factor of 0), and SNOMED CT disorder variables derived as described above.

Comparison of symptoms in patients with and without COVID-19

We used Cox proportional hazards models [6] to compare recording of symptoms after the index date in exposed and unexposed patients, analysing data for each symptom separately. The primary analysis was for the time period starting 12 weeks after the index date, i.e. the cut-off beyond which persistent symptoms may contribute to a Long Covid diagnosis according to World Health Organization (WHO) criteria [3]. Hazard ratios were adjusted for age, sex, age/sex interaction, number of consultations in the year before the index date, number of days on which any symptom was recorded 1-3 months before the index date, recording of the specific symptom 1-3 months before the index date, ethnicity, smoking and body mass index, stratified by general practice, with inverse probability of treatment weighting according to a generated propensity score generated as described above. We plotted Schoenfeld residuals to validate the proportional hazards assumption [6].

Clustering of Long Covid symptoms

We conducted a latent class analysis (LCA) of symptom prevalence among patients with confirmed COVID-19 and 'Long Covid' defined using two methods:

(a) For the main analysis, we defined 'Long Covid' as the presence of at least one symptom included in the WHO definition of post-COVID syndrome recorded beyond 12 weeks after the index date. We included all symptoms recorded in the 3 months after the first record of a WHO symptom in the latent class analysis, and excluded patients without a full 3-month follow up period after this date (to avoid bias in number of symptoms recorded based on duration of follow-up). We used the same set of symptoms for clustering as the study by Subramanian et al. [2] We used an elbow plot to identify the optimal number of classes.

(b) We also carried out a replication of the clustering analysis in the study by Subramanian et al. [2], defining 'Long Covid' as the presence of any of the 62 symptoms associated with COVID in that study, and without a time limit on when symptoms could be recorded.

We carried out analyses using the R statistical system (version 4.1), using the survival [6], glmnet [7] and and poLCA [8] packages.

Supplementary Results

Results of manual validation of information extracted from free text

Manual validation of text samples yielded precision estimates of 85-97% on the majority of information extraction tasks. Unfortunately a small number of text samples were erroneously truncated by the spreadsheet software used for manual review, so were unable to be used for validation.

We found that 507 / 583 (87.0%) of symptom mentions in the free text extracted by natural language processing were correct, with the majority of errors due to incorrect recognition of a hypothetical context in which symptoms were mentioned (e.g. 'Do not attend the surgery if you experience cough or fever'). The estimate of precision for symptoms from COVID-19 cases was 88.8% (261 / 294, 95% confidence interval (CI) 84.6%, 92.1%). The estimate of precision for symptoms from control patients was 85.1% (246 / 289, 95% CI 80.5%, 89.0%). There is no significant difference between these estimates ($p = 0.24$ by proportion test).

The phrase 'COVID-19' with context detected as ‘confirmed’ was recognised as a statement of COVID-19 diagnosis with 34.0% (95% CI 21.2%, 48.8%) precision. As with symptoms, the majority of errors were due to incorrect detection of context, such as reference to 'the Covid pandemic' or 'Covid precautions'. FMA assumes by default that a concept is a confirmed fact relating to the patient if no contextual information to the contrary is detected. We therefore ignored mentions of the phrase ‘COVID-19’ with ‘confirmed’ context extracted by NLP for patient classification, as they had a high risk of being incorrect.

FMA detected other COVID-19 related concepts with a good level of precision. Suspected COVID-19 (the phrase ‘COVID-19’ associated with phrases such as ‘suspected’, ‘possible’ or ‘query’) was detected with 86.9% precision (95% CI 82.0%, 90.9%). Precision for other specific acute COVID concepts (e.g. COVID pneumonia) was greater, at 95.1% (95% CI 91.6%, 97.5%) for confirmed concepts and 97.3% (93.1%, 99.2%) for suspected, and for chronic COVID-19 (ongoing symptomatic COVID-19 or post COVID-19 condition, suspected or confirmed) precision was 87.3% (82.4%, 91.3%).

Inter-rater reliability of the manual annotators was good for symptoms (unweighted kappa 0.75, 95% CI 0.66, 0.83) but moderate overall (weighted kappa 0.54, 95% CI 0.48, 0.61).

Supplementary References

1. Shah AD, Martinez C, Hemingway H. The freetext matching algorithm: a computer program to extract diagnoses and causes of death from unstructured text in electronic health records. BMC Med Inform Decis Mak 2012;12:88 doi: 10.1186/1472-6947-12-88 http://www.biomedcentral.com/1472-6947/12/88/

2. Subramanian A, Nirantharakumar K, Hughes S, et al. Symptoms and risk factors for long COVID in non-hospitalized adults. Nat Med 2022; published online July 25. DOI:10.1038/s41591-022-01909-w. https://www.nature.com/articles/s41591-022-01909-w

3. A clinical case definition of post COVID-19 condition by a Delphi consensus. World Health Organization, 2021 https://www.who.int/publications/i/item/WHO-2019-nCoV-Post_COVID-19_condition-Clinical_case_definition-2021.1

4. Elkheder M, Gonzalez-Izquierdo A, Qummer Ul Arfeen M, et al. Translating and evaluating historic phenotyping algorithms using SNOMED CT. J Am Med Inform Assoc 2023; 30(2): 222-232. https://doi.org/10.1093/jamia/ocac158

5. Tibshirani R. The Lasso Method for Variable Selection in the Cox Model. Statistics in Medicine 16(4): 385–95. https://doi.org/10.1002/(sici)1097-0258(19970228)16:4<385::aid-sim380>3.0.co;2-3

6. Therneau T. A Package for Survival Analysis in R. R package version 3.4-0, 2022. https://CRAN.R-project.org/package=survival

7. Friedman J, Hastie T, Tibshirani R. Regularization Paths for Generalized Linear Models via Coordinate Descent. Journal of Statistical Software 2010. 33(1), 1–22. doi:10.18637/jss.v033.i01, https://www.jstatsoft.org/v33/i01/.

8. Linzer DA, Lewis JB. poLCA: An R Package for Polytomous Variable Latent Class Analysis. Journal of Statistical Software 2011, 42(10), 1–29. https://www.jstatsoft.org/v42/i10/.
