# Supplementary material for: Long Covid symptoms and diagnosis in primary care: A cohort study using structured and unstructured data in The Health Improvement Network primary care database
Source: PLoS One. 2023 Sep 26;18(9):e0290583. doi: 10.1371/journal.pone.0290583 (PMC10521988; doi:10.1371/journal.pone.0290583)
Supplement: S3 Table — List of 98 symptoms investigated in this study, showing which symptoms are included in the WHO Long Covid case definition, which are used in the clustering analysis, and the percentage of days with either a free text or coded symptom record that the symptom is recorded using a coded entry. (PDF) [file pone.0290583.s005.pdf]

Supplementary Table S3: Recording of symptoms in free text and coded data

List of 98 symptoms investigated in this study, showing which symptoms are included in the WHO Long Covid case definition, which are used in the clustering analysis, and the percentage of days with either a free text or coded symptom record that the symptom is recorded using a coded entry.

Domain	Symptom	Number of Read terms in codelist	WHO case definition for Long Covid	Symptom in clustering analysis	Percentage (95% CI) of days with symptom record with a coded entry
Breathing	Shortness of breath	50	Y	Y	15.4 (15.1, 15.8)
	Wheezing	17		Y	3.7 (3.4, 4.1)
	Orthopnoea	4			8.7 (5.7, 12.7)
	Paroxysmal nocturnal dyspnoea	1			64.3 (35.1, 87.2)
Pain	Pain	500			18.2 (18.0, 18.4)
	Chest pain	68	Y	Y	23.9 (23.3, 24.6)
	Neuropathic pain	23			19.4 (18.5, 20.4)
Circulation	Presyncope / dizziness	14	Y	Y	19.4 (18.4, 20.3)
	Limb swelling	22		Y	17.4 (16.5, 18.3)
	Palpitations / tachycardia	34	Y	Y	22.5 (21.3, 23.7)
	Orthostatic hypotension	4			41.3 (37.2, 45.6)
	Cold extremities	7			5.4 (3.3, 8.3)
Fatigue	Fatigue / asthenia	78	Y	Y	12.4 (11.9, 12.9)
Cognitive health	Cognitive problems	95	Y	Y	39.9 (36.6, 43.4)
	Dysphasia	48			30.2 (22.3, 39.0)
	Dysarthria	7			14.1 (10.3, 18.7)
Movement	Tremors	15			12.7 (11.1, 14.4)
	Balance difficulty	13			7.6 (6.1, 9.2)
	Apraxia	57			51.2 (40.0, 62.3)
Sleep	Insomnia	30	Y	Y	38.4 (36.3, 40.6)
	Excessive sleep	8	Y		54.5 (23.4, 83.3)
Ear, nose and throat	Cough	23	Y	Y	19.0 (18.7, 19.4)
	Sore throat	31			44.0 (43.3, 44.6)
	Nasal congestion / sneezing	42		Y	21.0 (20.0, 22.0)
	Ear pain	12		Y	27.9 (26.6, 29.1)
	Phlegm	68		Y	8.2 (7.6, 8.8)
	Hearing loss	49	Y		34.6 (31.7, 37.6)
	Tinnitus	14			21.5 (19.3, 23.9)
	Dysphagia	22		Y	17.4 (15.9, 19.1)
	Hoarse voice	48		Y	11.5 (10.2, 12.9)
	Anosmia	10	Y	Y	11.2 (8.6, 14.4)
	Dysgeusia	6	Y		6.8 (5.1, 8.9)
	Hyperacusis	5	Y		12.5 (0.3, 52.7)
Stomach and digestion	Abdominal pain	45	Y	Y	27.9 (27.2, 28.6)
	Diarrhoea	72	Y	Y	19.9 (19.1, 20.7)
	Nausea / vomiting	106		Y	9.0 (8.6, 9.4)
	Constipation	22	Y	Y	18.8 (18.0, 19.6)

Domain	Symptom	Number of Read terms in codelist	WHO case definition for Long Covid	Symptom in clustering analysis	Percentage (95% CI) of days with symptom record with a coded entry
Stomach and digestion (contd.)	Gastric reflux	45	Y	Y	35.9 (34.6, 37.1)
	Weight loss	11		Y	7.4 (6.6, 8.1)
	Bloating	10		Y	10.7 (9.6, 11.9)
	Weight gain	5			3.8 (2.8, 5.0)
	Bowel incontinence	17		Y	62.7 (53.0, 71.8)
Muscles and joints	Joint pain	57	Y	Y	34.5 (33.6, 35.4)
	Muscle cramps	37	Y		13.0 (11.7, 14.3)
	Paraesthesia	12	Y	Y	7.3 (6.7, 7.9)
	Muscle pain	10	Y		12.5 (11.6, 13.6)
	Muscle twitch	9			11.1 (9.6, 12.7)
	Joint stiffmess	51			11.2 (7.4, 16.1)
Mental health	Anxiety / depression	199	Y	Y	32.1 (31.6, 32.6)
	Anorexia	9		Y	11.7 (9.8, 13.8)
	Mood swings	7			27.4 (24.1, 30.8)
	Post traumatic stress disorder	10			92.7 (87.3, 96.3)
	Loneliness	2			3.5 (1.9, 5.9)
	Increased appetite	1			1.4 (0.0, 7.6)
Hair, skin and nails	Purpura / rash	103		Y	12.0 (11.5, 12.4)
	Hives / itchy skin	57		Y	19.3 (18.1, 20.5)
	Nail changes	71		Y	55.5 (50.8, 60.1)
	Dry and scaly skin	20		Y	12.7 (11.2, 14.2)
	Hair loss	50		Y	29.8 (26.6, 33.1)
Eyes	Red / watery eye	109		Y	40.9 (38.2, 43.6)
	Dry eye	6		Y	38.4 (35.2, 41.7)
	Eye pain	3			16.4 (13.1, 20.2)
	Diplopia	8	Y		12.1 (9.8, 14.7)
	Itchy eyes	2			31.9 (23.6, 41.2)
	Flashing lights	4			76.9 (64.8, 86.5)
	Photophobia	1			1.5 (0.6, 3.0)
Reproductive health	Menorrhagia	14	Y	Y	35.1 (32.6, 37.6)
	Vaginal discharge	16		Y	28.1 (25.6, 30.6)
	Changes to menstrual period	23	Y		25.1 (22.3, 27.9)
	Sexual dysfunction	39		Y	59.7 (55.3, 63.9)
	Premenstrual syndrome	3	Y		89.4 (76.9, 96.5)
	Vaginal dryness	1			17.0 (12.2, 22.7)
Other symptoms	Allergies / angioedema	137	Y	Y	25.0 (23.9, 26.2)
	Headache	70	Y	Y	26.1 (25.4, 26.8)
	Chills and fever	56	Y	Y	13.1 (12.6, 13.6)
	Polyuria	13		Y	16.5 (15.3, 17.8)
	Vertigo	23		Y	30.3 (28.3, 32.3)
	Urinary incontinence	30		Y	30.9 (28.4, 33.6)
	Swelling of lymph nodes	39			10.6 (9.3, 11.9)

Domain	Symptom	Number of Read terms in codelist	WHO case definition for Long Covid	Symptom in clustering analysis	Percentage (95% CI) of days with symptom record with a coded entry
Other symptoms	Mouth ulcer	12		Y	63.0 (56.8, 68.8)
(contd.)	Hot flushes	4		Y	11.2 (9.2, 13.5)
	Sweating	15			7.2 (6.3, 8.2)
	Seizures	22			38.3 (35.2, 41.5)
	Body ache	3		Y	15.0 (12.8, 17.5)
	Haemoptysis	8		Y	12.2 (10.7, 13.9)
	Urinary retention	19		Y	28.7 (25.9, 31.6)
	Polydipsia	10			28.3 (22.0, 35.4)
	Dry mouth	10		Y	7.7 (6.2, 9.3)
	Hallucinations	26			23.2 (19.9, 26.8)
