# Supplementary material for: Long Covid symptoms and diagnosis in primary care: A cohort study using structured and unstructured data in The Health Improvement Network primary care database
Source: PLoS One. 2023 Sep 26;18(9):e0290583. doi: 10.1371/journal.pone.0290583 (PMC10521988; doi:10.1371/journal.pone.0290583)
Supplement: S4 Table — ‘Long Covid’ was defined as either (a) presence of any symptom included in the WHO case definition of post COVID condition at least 12 weeks after the initial COVID-19 diagnosis, or (b) a symptom included in the secondary outcome of the study by Subramanian et al. (Nat Med 2022, doi: 10.1038/s41591-022-01909-w, S3 Table), for comparison with that study. For (a), symptoms in the 3 months after the WHO symptom were used in the latent class analysis; for (b) symptom records at any time were used. Cluster descriptions for (a): Class 1 (81.2%): Shortness of breath (30%), Fatigue / asthenia (23%), Anxiety / depression (23%), Cough (18%), Joint pain (11%). Class 2 (18.8%): Shortness of breath (51%), Nausea / vomiting (45%), Anxiety / depression (41%), Cough (39%), Abdominal pain (36%), Chest pain (32%), Fatigue / asthenia (30%), Diarrhoea (25%), Constipation (25%), Chills and fever (23%), Purpura / rash (21%), Wheezing (19%), Headache (17%), Phlegm (16%), Palpitations / tachycardia (16%), Gastric reflux (13%), Limb swelling (13%), Presyncope / dizziness (13%), Paraesthesia (12%), Weight loss (12%), Bloating (11%), Joint pain (10%). Cluster descriptions for (b): Class 1 (78.2%): Shortness of breath (18%), Anxiety / depression (17%), Purpura / rash (14%), Fatigue / asthenia (14%). Class 2 (21.7%): Shortness of breath (63%), Cough (55%), Fatigue / asthenia (40%), Anxiety / depression (40%), Nausea / vomiting (34%), Chest pain (33%), Abdominal pain (27%), Wheezing (23%), Diarrhoea (21%), Constipation (20%), Phlegm (20%), Purpura / rash (20%), Chills and fever (19%), Headache (19%), Palpitations / tachycardia (16%), Limb swelling (15%), Presyncope / dizziness (15%), Joint pain (14%), Paraesthesia (14%), Gastric reflux (13%), Weight loss (12%). (PDF) [file pone.0290583.s006.pdf]

Supplementary Table S4: Two class latent class model for symptoms among patients with Long Covid

'Long Covid' was defined as either (a) presence of any symptom included in the WHO case definition of post COVID condition at least 12 weeks after the initial COVID-19 diagnosis, or (b) a symptom included in the secondary outcome of the study by Subramanian et al. (Nat Med 2022, doi:10.1038/s41591-022-01909-w, Supplementary Table 3), for comparison with that study. For (a), symptoms in the 3 months after the WHO symptom were used in the latent class analysis; for (b) symptom records at any time were used.

Cluster descriptions for (a):

Class 1 (81.2%): Shortness of breath (30%), Fatigue / asthenia (23%), Anxiety / depression (23%), Cough (18%), Joint pain (11%)

Class 2 (18.8%): Shortness of breath (51%), Nausea / vomiting (45%), Anxiety / depression (41%), Cough (39%), Abdominal pain (36%), Chest pain (32%), Fatigue / asthenia (30%), Diarrhoea (25%), Constipation (25%), Chills and fever (23%), Purpura / rash (21%), Wheezing (19%), Headache (17%), Phlegm (16%), Palpitations / tachycardia (16%), Gastric reflux (13%), Limb swelling (13%), Presyncope / dizziness (13%), Paraesthesia (12%), Weight loss (12%), Bloating (11%), Joint pain (10%)

Cluster descriptions for (b):

Class 1 (78.2%): Shortness of breath (18%), Anxiety / depression (17%), Purpura / rash (14%), Fatigue / asthenia (14%)

Class 2 (21.7%): Shortness of breath (63%), Cough (55%), Fatigue / asthenia (40%), Anxiety / depression (40%), Nausea / vomiting (34%), Chest pain (33%), Abdominal pain (27%), Wheezing (23%), Diarrhoea (21%), Constipation (20%), Phlegm (20%), Purpura / rash (20%), Chills and fever (19%), Headache (19%), Palpitations / tachycardia (16%), Limb swelling (15%), Presyncope / dizziness (15%), Joint pain (14%), Paraesthesia (14%), Gastric reflux (13%), Weight loss (12%)

		**(a) WHO definition of Long Covid, consistent time period (N = 1049)**		**(b) Replication of CPRD study (N = 1542)**	
	**Class and proportion of patients classified**	**Class 1 (0.812)**	**Class 2 (0.188)**	**Class 1 (0.782)**	**Class 2 (0.218)**
**Domain**	**Symptom**	**Item-response probabilities conditional on latent class membership**			
Breathing	Shortness of breath	0.302	0.511	0.177	0.626
	Wheezing	0.053	0.192	0.033	0.230
Pain	Chest pain	0.097	0.321	0.058	0.332
Circulation	Presyncope / dizziness	0.058	0.126	0.032	0.149
	Limb swelling	0.037	0.129	0.051	0.150
	Palpitations / tachycardia	0.049	0.159	0.029	0.159
Fatigue	Fatigue / asthenia	0.229	0.305	0.137	0.398
Cognitive health	Cognitive problems	0.022	0.000	0.016	0.012
Sleep	Insomnia	0.029	0.033	0.018	0.050
Ear, nose and throat	Cough	0.176	0.394	0.096	0.553
	Nasal congestion / sneezing	0.014	0.071	0.013	0.071
	Ear pain	0.010	0.052	0.014	0.050
	Phlegm	0.014	0.162	0.001	0.202
	Dysphagia	0.008	0.030	0.013	0.036
	Hoarse voice	0.008	0.027	0.004	0.032
	Anosmia	0.009	0.011	0.006	0.018
Stomach and digestion	Abdominal pain	0.087	0.361	0.079	0.268
	Diarrhoea	0.054	0.255	0.042	0.207
	Nausea / vomiting	0.062	0.447	0.092	0.343
	Constipation	0.069	0.248	0.054	0.203

		(a) WHO definition of Long Covid, consistent time period (N = 1049)		(b) Replication of CPRD study (N = 1542)	
	**Class and proportion of patients classified**	**Class 1 (0.812)**	**Class 2 (0.188)**	**Class 1 (0.782)**	**Class 2 (0.218)**
**Domain**	**Symptom**	**Item-response probabilities conditional on latent class membership**			
Stomach and digestion (contd.)	Gastric reflux	0.034	0.129	0.023	0.125
	Weight loss	0.030	0.121	0.047	0.124
	Bloating	0.009	0.112	0.013	0.085
	Bowel incontinence			0.002	0.004
Muscles and joints	Joint pain	0.106	0.105	0.074	0.145
	Paraesthesia	0.073	0.122	0.051	0.144
Mental health	Anxiety / depression	0.226	0.413	0.166	0.398
	Anorexia	0.002	0.027	0.004	0.028
Hair, skin and nails	Purpura / rash	0.061	0.208	0.139	0.198
	Hives / itchy skin	0.012	0.065	0.032	0.045
	Nail changes	0.000	0.010	0.001	0.009
	Dry and scaly skin	0.007	0.026	0.017	0.024
	Hair loss	0.021	0.019	0.027	0.033
Eyes	Red / watery eye	0.005	0.020	0.011	0.018
	Dry eye	0.005	0.038	0.008	0.041
Reproductive health	Menorrhagia	0.012	0.019	0.009	0.009
	Vaginal discharge	0.003	0.019	0.006	0.019
	Sexual dysfunction	0.004	0.005	0.004	0.005
Other symptoms	Allergies / angioedema	0.037	0.084	0.030	0.077
	Headache	0.085	0.166	0.064	0.186
	Chills and fever	0.033	0.226	0.028	0.193
	Polyuria	0.019	0.077	0.024	0.063
	Vertigo	0.013	0.050	0.014	0.048
	Urinary incontinence	0.012	0.035	0.012	0.037
	Mouth ulcer	0.001	0.012	0.003	0.007
	Hot flushes	0.004	0.022	0.006	0.013
	Body ache	0.003	0.034	0.004	0.029
	Haemoptysis	0.000	0.051	0.000	0.039
	Urinary retention	0.003	0.011	0.004	0.027
	Dry mouth	0.005	0.036	0.004	0.036
