# Supplementary material for: Long Covid symptoms and diagnosis in primary care: A cohort study using structured and unstructured data in The Health Improvement Network primary care database
Source: PLoS One. 2023 Sep 26;18(9):e0290583. doi: 10.1371/journal.pone.0290583 (PMC10521988; doi:10.1371/journal.pone.0290583)
Supplement: S5 Table — ‘Long Covid’ was defined as either (a) presence of any symptom included in the WHO case definition of post COVID condition at least 12 weeks after the initial COVID-19 diagnosis, or (b) a symptom included in the secondary outcome of the study by Subramanian et al. (Nat Med 2022, doi: 10.1038/s41591-022-01909-w, S3 Table), for comparison with that study. For (a), symptoms in the 3 months after the WHO symptom were used in the latent class analysis; for (b) symptom records at any time were used. Cluster descriptions for (a): Class 1 (50.8%): Anxiety / depression (26%), Fatigue / asthenia (19%), Abdominal pain (17%), Headache (13%), Nausea / vomiting (12%), Joint pain (11%), Diarrhoea (10%), Constipation (10%). Class 2 (35.2%): Shortness of breath (67%), Cough (34%), Fatigue / asthenia (28%), Chest pain (20%), Anxiety / depression (17%), Wheezing (13%). Class 3 (13.9%): Shortness of breath (64%), Anxiety / depression (49%), Nausea / vomiting (47%), Cough (42%), Chest pain (37%), Fatigue / asthenia (34%), Abdominal pain (34%), Constipation (28%), Diarrhoea (25%), Purpura / rash (24%), Wheezing (24%), Palpitations / tachycardia (22%), Chills and fever (21%), Headache (18%), Limb swelling (17%), Presyncope / dizziness (16%), Phlegm (16%), Gastric reflux (14%), Weight loss (14%), Paraesthesia (13%), Joint pain (13%), Bloating (12%), Allergies / angioedema (11%). Cluster descriptions for (b): Class 1 (68.8%): Anxiety / depression (17%), Purpura / rash (15%), Fatigue / asthenia (12%), Nausea / vomiting (10%), Shortness of breath (10%). Class 2 (19.0%): Shortness of breath (74%), Cough (56%), Chest pain (29%), Fatigue / asthenia (27%), Wheezing (27%), Anxiety / depression (21%), Phlegm (16%). Class 3 (12.3%): Shortness of breath (53%), Nausea / vomiting (53%), Anxiety / depression (50%), Fatigue / asthenia (47%), Abdominal pain (43%), Cough (41%), Diarrhoea (32%), Constipation (31%), Chest pain (30%), Purpura / rash (29%), Headache (26%), Chills and fever (24%), Presyncope / [file pone.0290583.s007.pdf]

Supplementary Table S5: Three class latent class model for symptoms among patients with Long Covid

'Long Covid' was defined as either (a) presence of any symptom included in the WHO case definition of post COVID condition at least 12 weeks after the initial COVID-19 diagnosis, or (b) a symptom included in the secondary outcome of the study by Subramanian et al. (Nat Med 2022, doi:10.1038/s41591-022-01909-w, Supplementary Table 3), for comparison with that study. For (a), symptoms in the 3 months after the WHO symptom were used in the latent class analysis; for (b) symptom records at any time were used.

Cluster descriptions for (a):

Class 1 (50.8%): Anxiety / depression (26%), Fatigue / asthenia (19%), Abdominal pain (17%), Headache (13%), Nausea / vomiting (12%), Joint pain (11%), Diarrhoea (10%), Constipation (10%)

Class 2 (35.2%): Shortness of breath (67%), Cough (34%), Fatigue / asthenia (28%), Chest pain (20%), Anxiety / depression (17%), Wheezing (13%)

Class 3 (13.9%): Shortness of breath (64%), Anxiety / depression (49%), Nausea / vomiting (47%), Cough (42%), Chest pain (37%), Fatigue / asthenia (34%), Abdominal pain (34%), Constipation (28%), Diarrhoea (25%), Purpura / rash (24%), Wheezing (24%), Palpitations / tachycardia (22%), Chills and fever (21%), Headache (18%), Limb swelling (17%), Presyncope / dizziness (16%), Phlegm (16%), Gastric reflux (14%), Weight loss (14%), Paraesthesia (13%), Joint pain (13%), Bloating (12%), Allergies / angioedema (11%)

Cluster descriptions for (b):

Class 1 (68.8%): Anxiety / depression (17%), Purpura / rash (15%), Fatigue / asthenia (12%), Nausea / vomiting (10%), Shortness of breath (10%)

Class 2 (19.0%): Shortness of breath (74%), Cough (56%), Chest pain (29%), Fatigue / asthenia (27%), Wheezing (27%), Anxiety / depression (21%), Phlegm (16%)

Class 3 (12.3%): Shortness of breath (53%), Nausea / vomiting (53%), Anxiety / depression (50%), Fatigue / asthenia (47%), Abdominal pain (43%), Cough (41%), Diarrhoea (32%), Constipation (31%), Chest pain (30%), Purpura / rash (29%), Headache (26%), Chills and fever (24%), Presyncope / dizziness (22%), Palpitations / tachycardia (21%), Paraesthesia (20%), Joint pain (18%), Weight loss (18%), Limb swelling (18%), Gastric reflux (18%), Bloating (15%), Wheezing (14%), Phlegm (12%)

		**(a) WHO definition of Long Covid, consistent time period (N = 1049)**			**(b) Replication of CPRD study (N = 1542)**		
	**Class and proportion of patients classified**	**Class 1 (0.508)**	**Class 2 (0.353)**	**Class 3 (0.139)**	**Class 1 (0.688)**	**Class 2 (0.190)**	**Class 3 (0.123)**
**Domain**	**Symptom**	**Item-response probabilities conditional on latent class membership**					
Breathing	Shortness of breath	0.029	0.672	0.644	0.101	0.741	0.526
	Wheezing	0.000	0.129	0.241	0.011	0.271	0.139
Pain	Chest pain	0.036	0.198	0.368	0.035	0.295	0.304
Circulation	Presyncope / dizziness	0.064	0.043	0.165	0.034	0.043	0.216
	Limb swelling	0.011	0.072	0.168	0.046	0.099	0.179
	Palpitations / tachycardia	0.028	0.070	0.219	0.026	0.069	0.212
Fatigue	Fatigue / asthenia	0.190	0.281	0.340	0.124	0.272	0.466
Cognitive health	Cognitive problems	0.021	0.021	0.000	0.013	0.022	0.016
Sleep	Insomnia	0.036	0.015	0.043	0.017	0.031	0.064
Ear, nose and throat	Cough	0.076	0.342	0.417	0.057	0.558	0.408
	Nasal congestion / sneezing	0.022	0.006	0.082	0.014	0.044	0.063
	Ear pain	0.016	0.004	0.062	0.016	0.004	0.082
	Phlegm	0.000	0.058	0.156	0.000	0.159	0.119
	Dysphagia	0.006	0.012	0.037	0.013	0.021	0.044
	Hoarse voice	0.007	0.011	0.028	0.003	0.014	0.044

		(a) WHO definition of Long Covid, consistent time period (N = 1049)			(b) Replication of CPRD study (N = 1542)		
	**Class and proportion of patients classified**	**Class 1 (0.508)**	**Class 2 (0.353)**	**Class 3 (0.139)**	**Class 1 (0.688)**	**Class 2 (0.190)**	**Class 3 (0.123)**
**Domain**	**Symptom**	**Item-response probabilities conditional on latent class membership**					
	Anosmia	0.011	0.011	0.000	0.005	0.015	0.017
Stomach and digestion	Abdominal pain	0.172	0.012	0.335	0.088	0.036	0.431
	Diarrhoea	0.103	0.012	0.250	0.048	0.033	0.316
	Nausea / vomiting	0.121	0.021	0.471	0.101	0.067	0.525
	Constipation	0.102	0.033	0.284	0.059	0.039	0.312
	Gastric reflux	0.044	0.025	0.145	0.024	0.038	0.176
	Weight loss	0.041	0.020	0.137	0.051	0.032	0.182
	Bloating	0.019	0.005	0.122	0.015	0.000	0.151
	Bowel incontinence	-	-	-	0.002	0.003	0.005
Muscles and joints	Joint pain	0.113	0.087	0.126	0.075	0.079	0.185
	Paraesthesia	0.098	0.040	0.130	0.053	0.053	0.201
Mental health	Anxiety / depression	0.259	0.174	0.489	0.168	0.212	0.498
	Anorexia	0.005	0.000	0.031	0.004	0.000	0.049
Hair, skin and nails	Purpura / rash	0.095	0.020	0.241	0.153	0.059	0.289
	Hives / itchy skin	0.016	0.007	0.080	0.035	0.020	0.061
	Nail changes	0.000	0.000	0.014	0.001	0.000	0.016
	Dry and scaly skin	0.012	0.000	0.033	0.019	0.006	0.035
	Hair loss	0.019	0.024	0.021	0.030	0.009	0.047
Eyes	Red / watery eye	0.007	0.000	0.028	0.012	0.007	0.024
	Dry eye	0.010	0.001	0.042	0.010	0.005	0.065
Reproductive health	Menorrhagia	0.019	0.002	0.021	0.010	0.000	0.018
	Vaginal discharge	0.007	0.000	0.014	0.007	0.004	0.028
	Sexual dysfunction	0.006	0.002	0.000	0.004	0.005	0.004
Other symptoms	Allergies / angioedema	0.047	0.018	0.112	0.029	0.058	0.075
	Headache	0.132	0.023	0.181	0.067	0.064	0.264
	Chills and fever	0.058	0.029	0.214	0.029	0.078	0.237
	Polyuria	0.024	0.023	0.067	0.025	0.032	0.076
	Vertigo	0.025	0.000	0.054	0.016	0.000	0.084
	Urinary incontinence	0.014	0.012	0.037	0.011	0.028	0.039
	Mouth ulcer	0.000	0.003	0.014	0.002	0.006	0.011
	Hot flushes	0.009	0.000	0.022	0.006	0.008	0.018
	Body ache	0.008	0.000	0.034	0.005	0.000	0.051
	Haemoptysis	0.000	0.006	0.053	0.000	0.034	0.016
	Urinary retention	0.002	0.004	0.015	0.004	0.010	0.037
	Dry mouth	0.006	0.015	0.015	0.003	0.023	0.038
