# Supplementary figures and images for: Long Covid symptoms and diagnosis in primary care: A cohort study using structured and unstructured data in The Health Improvement Network primary care database
Source: PLoS One. 2023 Sep 26;18(9):e0290583. doi: 10.1371/journal.pone.0290583 (PMC10521988; doi:10.1371/journal.pone.0290583)

Supplementary Figure S1: Data flow diagram

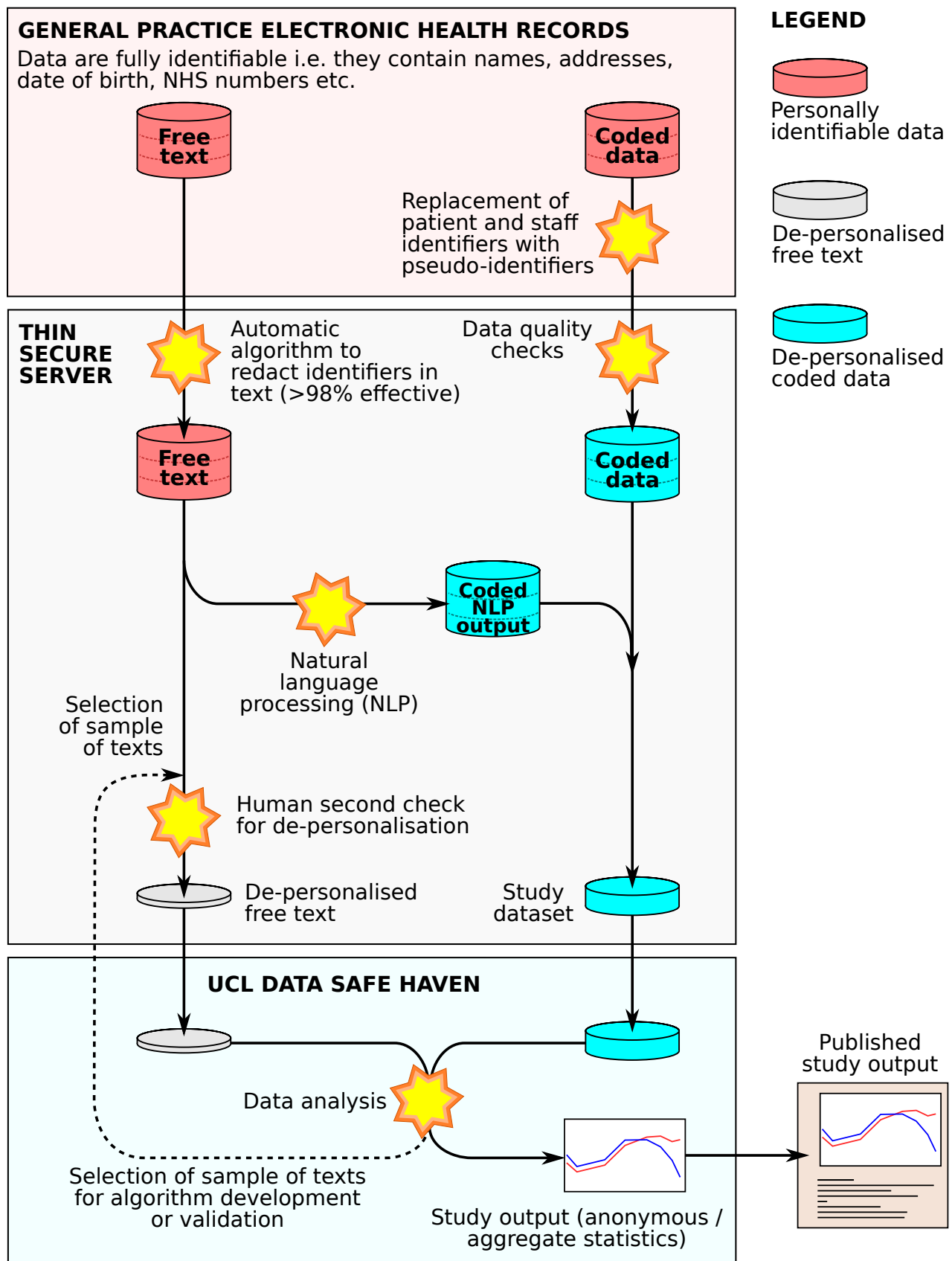

Supplement: S1 Fig — (PDF) [file pone.0290583.s008.pdf]
