# Supplementary material for: Long Covid symptoms and diagnosis in primary care: A cohort study using structured and unstructured data in The Health Improvement Network primary care database
Source: PLoS One. 2023 Sep 26;18(9):e0290583. doi: 10.1371/journal.pone.0290583 (PMC10521988; doi:10.1371/journal.pone.0290583)
Supplement: S2 Fig — Association of symptoms with previous COVID infection after 12 weeks. Hazard ratios were adjusted for age, sex, age/sex interaction, number of consultations in the year before the index date, number of symptom days 1–3 months before the index date, recording of the specific symptom 1–3 months before the index date, ethnicity, smoking, body mass index and a generated propensity score for acquiring COVID-19 infection, and stratified by general practice. (PDF) [file pone.0290583.s009.pdf]

Supplementary Figure S2: Hazard ratios for all 89 symptoms

Association of symptoms with previous COVID infection after 12 weeks. Hazard ratios were adjusted for age, sex, age/sex interaction, number of consultations in the year before the index date, number of symptom days 1-3 months before the index date, recording of the specific symptom 1-3 months before the index date, ethnicity, smoking, body mass index and a generated propensity score for acquiring COVID-19 infection, and stratified by general practice.

Symptom by domain	N events	Hazard ratio for symptom in confirmed COVID-19 versus unexposed
Breathing		
Orthopnoea	14	1.25 (0.27, 5.87)
Paroxysmal nocturnal dyspnoea	0	–
Shortness of breath	871	2.89 (2.48, 3.36)
Wheezing	241	2.13 (1.58, 2.88)
Pain		
Chest pain	420	2.13 (1.70, 2.67)
Neuropathic pain	285	1.46 (1.09, 1.94)
Pain	3439	1.29 (1.19, 1.40)
Circulation		
Cold extremities	16	0.63 (0.16, 2.52)
Limb swelling	280	1.83 (1.38, 2.43)
Orthostatic hypotension	29	0.76 (0.26, 2.19)
Palpitations / tachycardia	190	2.59 (1.86, 3.60)
Presyncope / dizziness	298	1.29 (0.98, 1.71)
Fatigue		
Fatigue / asthenia	606	3.46 (2.87, 4.17)
Cognitive health		
Cognitive problems	63	1.88 (1.05, 3.35)
Dysarthria	14	2.84 (0.71, 11.35)
Dysphasia	8	–
Movement		
Apraxia	3	0.65 (0.00, Inf)
Balance difficulty	74	1.40 (0.78, 2.51)
Tremors	61	1.26 (0.67, 2.37)
Sleep		
Excessive sleep	0	–
Insomnia	82	1.89 (1.19, 3.01)
Ear, nose and throat		
Anosmia	18	11.34 (2.88, 44.69)
Cough	662	2.08 (1.74, 2.48)
Dysgeusia	27	0.93 (0.34, 2.60)
Dysphagia	72	1.65 (0.96, 2.85)
Ear pain	134	1.04 (0.67, 1.63)
Hearing loss	56	0.94 (0.48, 1.82)
Hoarse voice	38	1.78 (0.81, 3.89)
Hyperacusis	0	–
Nasal congestion / sneezing	134	1.16 (0.76, 1.77)
Phlegm	138	2.43 (1.65, 3.59)
Sore throat	165	1.61 (1.12, 2.31)
Tinnitus	58	0.96 (0.48, 1.89)
Stomach and digestion		
Abdominal pain	552	1.42 (1.15, 1.74)
Bloating	140	1.31 (0.87, 1.98)
Bowel incontinence	5	–
Constipation	391	1.26 (0.99, 1.61)
Diarrhoea	329	1.50 (1.15, 1.95)
Gastric reflux	200	1.44 (1.03, 2.00)
Nausea / vomiting	588	1.76 (1.45, 2.13)
Weight gain	75	2.73 (1.60, 4.66)
Weight loss	286	1.47 (1.11, 1.96)

Hazard ratio (axis: 1, 2, 3, 4, 5)

Symptom by domain	N events	Hazard ratio for symptom in confirmed COVID-19 versus unexposed
Muscles and joints		
Joint pain	488	1.06 (0.85, 1.33)
Joint stiffmess	24	1.00 (0.33, 3.08)
Muscle cramps	121	1.42 (0.92, 2.20)
Muscle pain	151	1.86 (1.26, 2.75)
Muscle twitch	57	1.13 (0.58, 2.19)
Paraesthesia	318	1.68 (1.29, 2.18)
Mental health		
Anorexia	42	1.21 (0.57, 2.54)
Anxiety / depression	988	1.55 (1.33, 1.81)
Increased appetite	7	5.67 (0.87, 37.06)
Loneliness	14	2.02 (0.57, 7.15)
Mood swings	27	1.12 (0.35, 3.58)
Post traumatic stress disorder	6	1.36 (0.17, 10.90)
Hair, skin and nails		
Dry and scaly skin	105	0.74 (0.44, 1.24)
Hair loss	64	8.18 (4.54, 14.75)
Hives / itchy skin	182	1.26 (0.87, 1.82)
Nail changes	22	0.48 (0.13, 1.80)
Purpura / rash	841	1.22 (1.03, 1.45)
Eyes		
Diplopia	36	1.05 (0.45, 2.45)
Dry eye	58	1.80 (0.96, 3.37)
Eye pain	24	2.21 (0.73, 6.63)
Flashing lights	4	–
Itchy eyes	6	–
Photophobia	21	4.02 (1.44, 11.16)
Red / watery eye	59	1.45 (0.74, 2.83)
Reproductive health		
Changes to menstrual period	58	0.90 (0.43, 1.88)
Menorrhagia	74	1.05 (0.56, 1.96)
Premenstrual syndrome	0	–
Sexual dysfunction	23	0.85 (0.26, 2.76)
Vaginal discharge	65	1.04 (0.54, 2.00)
Vaginal dryness	12	1.91 (0.24, 15.22)
Other symptoms		
Allergies / angioedema	188	1.24 (0.87, 1.77)
Body ache	28	1.68 (0.72, 3.90)
Chills and fever	279	1.42 (1.07, 1.87)
Dry mouth	48	1.24 (0.65, 2.36)
Haemoptysis	28	1.97 (0.83, 4.68)
Hallucinations	29	1.87 (0.84, 4.15)
Headache	399	1.77 (1.39, 2.25)
Hot flushes	48	1.11 (0.54, 2.26)
Mouth ulcer	9	3.02 (0.52, 17.52)
Polydipsia	6	–
Polyuria	179	0.90 (0.61, 1.31)
Seizures	30	3.48 (1.34, 9.07)
Sweating	100	1.48 (0.90, 2.44)
Swelling of lymph nodes	35	1.28 (0.53, 3.08)
Urinary incontinence	75	1.34 (0.77, 2.33)
Urinary retention	44	1.13 (0.52, 2.46)
Vertigo	107	1.21 (0.75, 1.93)

Hazard ratio (axis: 1, 2, 3, 4, 5)
