# Supplementary material for: Long Covid symptoms and diagnosis in primary care: A cohort study using structured and unstructured data in The Health Improvement Network primary care database
Source: PLoS One. 2023 Sep 26;18(9):e0290583. doi: 10.1371/journal.pone.0290583 (PMC10521988; doi:10.1371/journal.pone.0290583)

Supplementary Figure S3: Hazard ratios by level of adjustment

Association of symptoms with previous COVID infection after 12 weeks, by level of adjustment. 'Fully adjusted hazard ratios' were adjusted for age, sex, age/sex interaction, number of consultations in the year before the index date, number of symptom days 1-3 months before the index date, recording of the specific symptom 1-3 months before the index date, ethnicity, smoking, body mass index and a generated propensity score for acquiring COVID-19 infection, and stratified by general practice.

Symptom by domain	N events	Hazard ratio for symptom in confirmed COVID-19 versus unexposed
Breathing		
**Shortness of breath**		
Fully adjusted	871	2.89 (2.48, 3.36)
Crude	871	3.80 (3.33, 4.34)
**Wheezing**		
Fully adjusted	241	2.13 (1.58, 2.88)
Crude	241	3.62 (2.81, 4.67)
Pain		
**Chest pain**		
Fully adjusted	420	2.13 (1.70, 2.67)
Crude	420	2.84 (2.34, 3.44)
**Neuropathic pain**		
Fully adjusted	285	1.46 (1.09, 1.94)
Crude	285	1.80 (1.40, 2.30)
**Pain**		
Fully adjusted	3439	1.29 (1.19, 1.40)
Crude	3439	1.69 (1.57, 1.82)
Circulation		
**Limb swelling**		
Fully adjusted	280	1.83 (1.38, 2.43)
Crude	280	2.45 (1.93, 3.11)
**Palpitations / tachycardia**		
Fully adjusted	190	2.59 (1.86, 3.60)
Crude	190	3.27 (2.46, 4.35)
**Presyncope / dizziness**		
Fully adjusted	298	1.29 (0.98, 1.71)
Crude	298	1.60 (1.25, 2.06)
Fatigue		
**Fatigue / asthenia**		
Fully adjusted	606	3.46 (2.87, 4.17)
Crude	606	3.83 (3.26, 4.49)
Ear, nose and throat		
**Cough**		
Fully adjusted	662	2.08 (1.74, 2.48)
Crude	662	3.31 (2.84, 3.86)
**Ear pain**		
Fully adjusted	134	1.04 (0.67, 1.63)
Crude	134	1.28 (0.86, 1.88)
**Nasal congestion / sneezing**		
Fully adjusted	134	1.16 (0.76, 1.77)
Crude	134	1.59 (1.10, 2.31)
**Phlegm**		
Fully adjusted	138	2.43 (1.65, 3.59)
Crude	138	3.81 (2.73, 5.32)
**Sore throat**		
Fully adjusted	165	1.61 (1.12, 2.31)
Crude	165	2.04 (1.48, 2.81)
Stomach and digestion		
**Abdominal pain**		
Fully adjusted	552	1.42 (1.15, 1.74)
Crude	552	1.89 (1.58, 2.25)

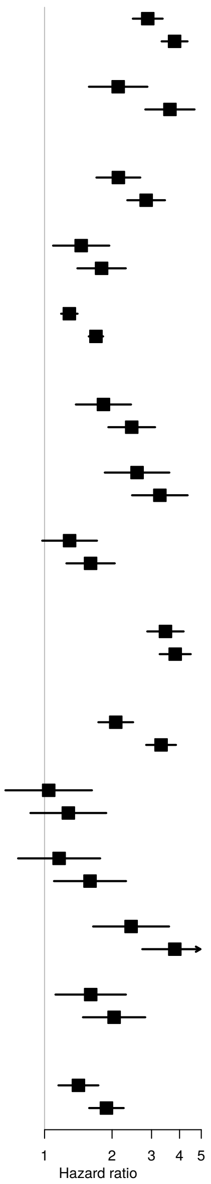

Symptom by domain	N events	Hazard ratio for symptom in confirmed COVID-19 versus unexposed
**Constipation**		
Fully adjusted	391	1.26 (0.99, 1.61)
Crude	391	1.96 (1.59, 2.41)
**Diarrhoea**		
Fully adjusted	329	1.50 (1.15, 1.95)
Crude	329	2.16 (1.72, 2.70)
**Gastric reflux**		
Fully adjusted	200	1.44 (1.03, 2.00)
Crude	200	1.99 (1.49, 2.67)
**Nausea / vomiting**		
Fully adjusted	588	1.76 (1.45, 2.13)
Crude	588	2.37 (2.01, 2.80)
**Weight loss**		
Fully adjusted	286	1.47 (1.11, 1.96)
Crude	286	1.99 (1.56, 2.54)
Muscles and joints		
**Joint pain**		
Fully adjusted	488	1.06 (0.85, 1.33)
Crude	488	1.50 (1.23, 1.82)
**Muscle pain**		
Fully adjusted	151	1.86 (1.26, 2.75)
Crude	151	2.55 (1.85, 3.54)
**Paraesthesia**		
Fully adjusted	318	1.68 (1.29, 2.18)
Crude	318	1.98 (1.57, 2.50)
Mental health		
**Anxiety / depression**		
Fully adjusted	988	1.55 (1.33, 1.81)
Crude	988	1.99 (1.75, 2.28)
Hair, skin and nails		
**Hives / itchy skin**		
Fully adjusted	182	1.26 (0.87, 1.82)
Crude	182	1.58 (1.15, 2.17)
**Purpura / rash**		
Fully adjusted	841	1.22 (1.03, 1.45)
Crude	841	1.46 (1.26, 1.70)
Other symptoms		
**Allergies / angioedema**		
Fully adjusted	188	1.24 (0.87, 1.77)
Crude	188	1.81 (1.33, 2.45)
**Chills and fever**		
Fully adjusted	279	1.42 (1.07, 1.87)
Crude	279	2.01 (1.57, 2.58)
**Headache**		
Fully adjusted	399	1.77 (1.39, 2.25)
Crude	399	2.01 (1.64, 2.47)
**Polyuria**		
Fully adjusted	179	0.90 (0.61, 1.31)
Crude	179	1.45 (1.05, 2.01)

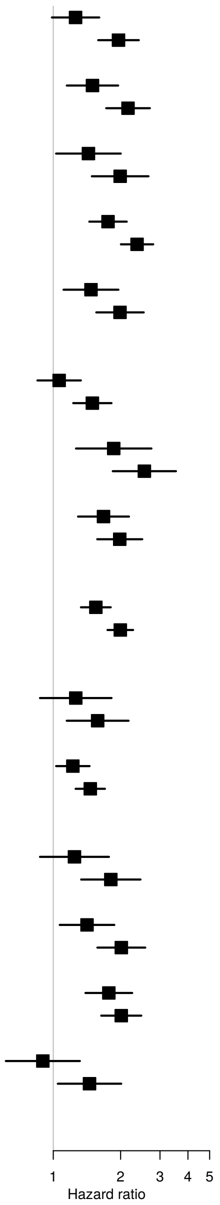

Supplement: S3 Fig — Association of symptoms with previous COVID infection after 12 weeks, by level of adjustment. ‘Fully adjusted hazard ratios’ were adjusted for age, sex, age/sex interaction, number of consultations in the year before the index date, number of symptom days 1–3 months before the index date, recording of the specific symptom 1–3 months before the index date, ethnicity, smoking, body mass index and a generated propensity score for acquiring COVID-19 infection, and stratified by general practice. (PDF) [file pone.0290583.s010.pdf]
