# Supplementary material for: Long Covid symptoms and diagnosis in primary care: A cohort study using structured and unstructured data in The Health Improvement Network primary care database
Source: PLoS One. 2023 Sep 26;18(9):e0290583. doi: 10.1371/journal.pone.0290583 (PMC10521988; doi:10.1371/journal.pone.0290583)
Supplement: S4 Fig — Association of symptoms with previous infection after 12 weeks, by age. Hazard ratios were adjusted for age, sex, age/sex interaction, number of consultations in the year before the index date, number of symptom days 1–3 months before the index date, recording of the specific symptom 1–3 months before the index date, ethnicity, smoking, body mass index and a generated propensity score for acquiring COVID-19 infection, and stratified by general practice. (PDF) [file pone.0290583.s011.pdf]

Supplementary Figure S4: Hazard ratios by age

Association of symptoms with previous infection after 12 weeks, by age. Hazard ratios were adjusted for age, sex, age/sex interaction, number of consultations in the year before the index date, number of symptom days 1-3 months before the index date, recording of the specific symptom 1-3 months before the index date, ethnicity, smoking, body mass index and a generated propensity score for acquiring COVID-19 infection, and stratified by general practice.

Symptom by domain	N events	Hazard ratio for symptom in confirmed COVID-19 versus unexposed
Breathing		
**Shortness of breath**		
Age < 50	235	4.16 (3.24, 5.33)
Age 50+	636	2.52 (2.12, 2.99)
**Wheezing**		
Age < 50	82	3.14 (2.01, 4.91)
Age 50+	159	1.75 (1.23, 2.49)
Pain		
**Chest pain**		
Age < 50	151	3.01 (2.19, 4.12)
Age 50+	269	1.72 (1.31, 2.26)
**Neuropathic pain**		
Age < 50	92	1.71 (1.09, 2.68)
Age 50+	193	1.35 (0.96, 1.89)
**Pain**		
Age < 50	1249	1.41 (1.24, 1.60)
Age 50+	2190	1.23 (1.11, 1.36)
Circulation		
**Limb swelling**		
Age < 50	34	2.64 (1.40, 4.96)
Age 50+	246	1.73 (1.28, 2.33)
**Palpitations / tachycardia**		
Age < 50	77	3.41 (2.18, 5.32)
Age 50+	113	2.10 (1.40, 3.17)
**Presyncope / dizziness**		
Age < 50	95	2.10 (1.39, 3.18)
Age 50+	203	1.00 (0.71, 1.41)
Fatigue		
**Fatigue / asthenia**		
Age < 50	234	3.32 (2.55, 4.32)
Age 50+	372	3.55 (2.85, 4.43)
Ear, nose and throat		
**Cough**		
Age < 50	190	2.18 (1.64, 2.89)
Age 50+	472	2.04 (1.66, 2.49)
**Ear pain**		
Age < 50	66	1.09 (0.59, 2.00)
Age 50+	68	1.01 (0.57, 1.78)
**Nasal congestion / sneezing**		
Age < 50	55	1.13 (0.61, 2.06)
Age 50+	79	1.19 (0.71, 2.00)
**Phlegm**		
Age < 50	35	2.86 (1.51, 5.43)
Age 50+	103	2.29 (1.48, 3.54)
**Sore throat**		
Age < 50	84	1.68 (1.03, 2.71)
Age 50+	81	1.54 (0.97, 2.47)
Stomach and digestion		
**Abdominal pain**		
Age < 50	236	1.73 (1.31, 2.28)
Age 50+	316	1.21 (0.93, 1.57)
**Constipation**		
Age < 50	112	1.52 (1.00, 2.30)
Age 50+	279	1.17 (0.89, 1.55)
**Diarrhoea**		
Age < 50	88	1.67 (1.07, 2.58)
Age 50+	241	1.44 (1.06, 1.94)
**Gastric reflux**		
Age < 50	76	1.62 (1.01, 2.60)
Age 50+	124	1.32 (0.88, 1.99)
**Nausea / vomiting**		
Age < 50	221	1.62 (1.22, 2.15)
Age 50+	367	1.85 (1.47, 2.33)
**Weight loss**		
Age < 50	74	1.53 (0.93, 2.51)
Age 50+	212	1.46 (1.06, 2.00)
Muscles and joints		
**Joint pain**		
Age < 50	141	1.24 (0.87, 1.77)
Age 50+	347	0.99 (0.76, 1.28)
**Muscle pain**		
Age < 50	55	2.69 (1.55, 4.67)
Age 50+	96	1.49 (0.93, 2.38)
**Paraesthesia**		
Age < 50	131	2.19 (1.53, 3.14)
Age 50+	187	1.38 (0.99, 1.92)
Mental health		
**Anxiety / depression**		
Age < 50	475	1.64 (1.34, 2.02)
Age 50+	513	1.47 (1.21, 1.79)
Hair, skin and nails		
**Hives / itchy skin**		
Age < 50	59	1.32 (0.73, 2.39)
Age 50+	123	1.23 (0.80, 1.89)
**Purpura / rash**		
Age < 50	413	1.45 (1.15, 1.82)
Age 50+	428	1.06 (0.85, 1.33)
Other symptoms		
**Allergies / angioedema**		
Age < 50	78	1.50 (0.91, 2.47)
Age 50+	110	1.09 (0.70, 1.70)
**Chills and fever**		
Age < 50	95	1.48 (0.96, 2.27)
Age 50+	184	1.38 (0.99, 1.93)
**Headache**		
Age < 50	195	1.97 (1.44, 2.70)
Age 50+	204	1.61 (1.18, 2.19)
**Polyuria**		
Age < 50	48	0.98 (0.49, 1.95)
Age 50+	131	0.87 (0.57, 1.34)

Hazard ratio (axis: 1, 2, 3, 4, 5)
