# Supplementary material for: Long Covid symptoms and diagnosis in primary care: A cohort study using structured and unstructured data in The Health Improvement Network primary care database
Source: PLoS One. 2023 Sep 26;18(9):e0290583. doi: 10.1371/journal.pone.0290583 (PMC10521988; doi:10.1371/journal.pone.0290583)

Supplementary Figure S5: Hazard ratios by sex

Association of symptoms with previous infection after 12 weeks, by sex. Hazard ratios were adjusted for age, sex, age/sex interaction, number of consultations in the year before the index date, number of symptom days 1-3 months before the index date, recording of the specific symptom 1-3 months before the index date, ethnicity, smoking, body mass index and a generated propensity score for acquiring COVID-19 infection, and stratified by general practice.

Symptom by domain	N events	Hazard ratio for symptom in confirmed COVID-19 versus unexposed
Breathing		
**Shortness of breath**		
Male	330	2.58 (2.05, 3.26)
Female	541	3.09 (2.56, 3.72)
**Wheezing**		
Male	84	1.24 (0.76, 2.01)
Female	157	2.87 (1.99, 4.12)
Pain		
**Chest pain**		
Male	149	1.72 (1.20, 2.45)
Female	271	2.41 (1.84, 3.15)
**Neuropathic pain**		
Male	88	0.96 (0.58, 1.58)
Female	197	1.76 (1.26, 2.46)
**Pain**		
Male	1016	1.25 (1.08, 1.44)
Female	2423	1.31 (1.19, 1.44)
Circulation		
**Limb swelling**		
Male	89	2.10 (1.32, 3.33)
Female	191	1.72 (1.23, 2.39)
**Palpitations / tachycardia**		
Male	47	3.99 (2.16, 7.37)
Female	143	2.23 (1.53, 3.25)
**Presyncope / dizziness**		
Male	96	1.05 (0.65, 1.69)
Female	202	1.42 (1.03, 1.98)
Fatigue		
**Fatigue / asthenia**		
Male	165	4.37 (3.16, 6.04)
Female	441	3.16 (2.55, 3.91)
Ear, nose and throat		
**Cough**		
Male	241	1.90 (1.45, 2.50)
Female	421	2.19 (1.76, 2.72)
**Ear pain**		
Male	25	1.08 (0.43, 2.71)
Female	109	1.04 (0.64, 1.69)
**Nasal congestion / sneezing**		
Male	36	1.37 (0.66, 2.81)
Female	98	1.09 (0.67, 1.77)
**Phlegm**		
Male	58	1.71 (0.97, 3.01)
Female	80	3.19 (1.93, 5.26)
**Sore throat**		
Male	36	1.11 (0.52, 2.35)
Female	129	1.78 (1.19, 2.65)
Stomach and digestion		
**Abdominal pain**		
Male	133	1.44 (0.99, 2.10)
Female	419	1.41 (1.12, 1.77)

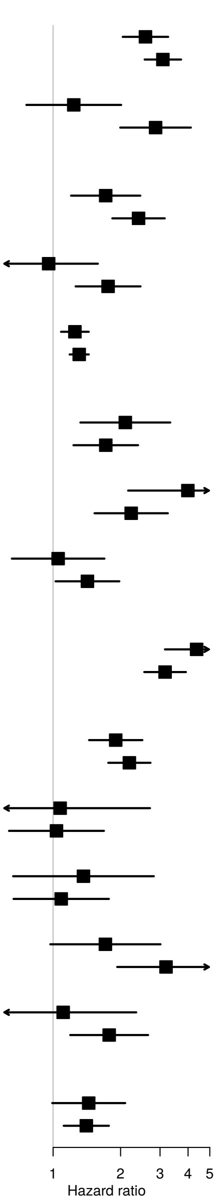

Symptom by domain	N events	Hazard ratio for symptom in confirmed COVID-19 versus unexposed
**Constipation**		
Male	118	1.56 (1.05, 2.33)
Female	273	1.14 (0.85, 1.52)
**Diarrhoea**		
Male	103	1.70 (1.11, 2.59)
Female	226	1.41 (1.03, 1.93)
**Gastric reflux**		
Male	63	1.29 (0.73, 2.26)
Female	137	1.51 (1.02, 2.23)
**Nausea / vomiting**		
Male	151	2.34 (1.66, 3.29)
Female	437	1.58 (1.27, 1.98)
**Weight loss**		
Male	113	1.45 (0.96, 2.20)
Female	173	1.49 (1.05, 2.11)
Muscles and joints		
**Joint pain**		
Male	164	1.11 (0.78, 1.58)
Female	324	1.04 (0.79, 1.36)
**Muscle pain**		
Male	28	1.15 (0.50, 2.65)
Female	123	2.09 (1.37, 3.18)
**Paraesthesia**		
Male	104	1.52 (0.98, 2.35)
Female	214	1.76 (1.29, 2.40)
Mental health		
**Anxiety / depression**		
Male	268	1.40 (1.07, 1.83)
Female	720	1.61 (1.35, 1.92)
Hair, skin and nails		
**Hives / itchy skin**		
Male	61	1.56 (0.88, 2.76)
Female	121	1.13 (0.72, 1.75)
**Purpura / rash**		
Male	196	1.20 (0.87, 1.65)
Female	645	1.23 (1.01, 1.50)
Other symptoms		
**Allergies / angioedema**		
Male	48	0.99 (0.52, 1.91)
Female	140	1.35 (0.90, 2.01)
**Chills and fever**		
Male	96	1.54 (0.99, 2.40)
Female	183	1.35 (0.96, 1.90)
**Headache**		
Male	93	1.67 (1.07, 2.61)
Female	306	1.81 (1.38, 2.36)
**Polyuria**		
Male	74	0.76 (0.43, 1.34)
Female	105	1.01 (0.63, 1.62)

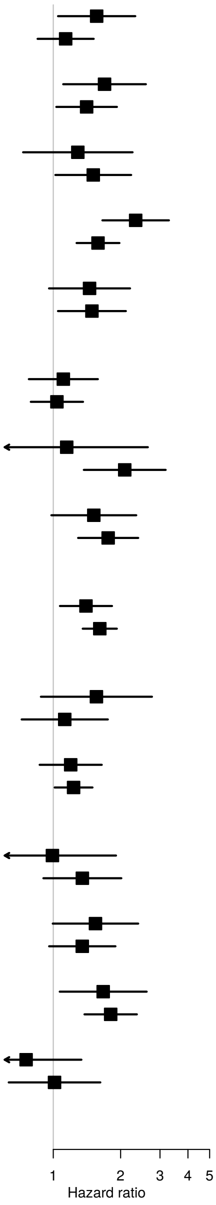

Supplement: S5 Fig — Association of symptoms with previous infection after 12 weeks, by sex. Hazard ratios were adjusted for age, sex, age/sex interaction, number of consultations in the year before the index date, number of symptom days 1–3 months before the index date, recording of the specific symptom 1–3 months before the index date, ethnicity, smoking, body mass index and a generated propensity score for acquiring COVID-19 infection, and stratified by general practice. (PDF) [file pone.0290583.s012.pdf]
