# Supplementary material for: Long Covid symptoms and diagnosis in primary care: A cohort study using structured and unstructured data in The Health Improvement Network primary care database
Source: PLoS One. 2023 Sep 26;18(9):e0290583. doi: 10.1371/journal.pone.0290583 (PMC10521988; doi:10.1371/journal.pone.0290583)

Supplementary Figure S6: Hazard ratios by nation

Association of symptoms with previous infection after 12 weeks, by UK nation. Hazard ratios were adjusted for age, sex, age/sex interaction, number of consultations in the year before the index date, number of symptom days 1-3 months before the index date, recording of the specific symptom 1-3 months before the index date, ethnicity, smoking, body mass index and a generated propensity score for acquiring COVID-19 infection, and stratified by general practice.

Symptom by domain	N events	Hazard ratio for symptom in confirmed COVID-19 versus unexposed
Breathing		
**Shortness of breath**		
England	211	2.66 (1.98, 3.56)
Scotland	338	2.44 (1.92, 3.10)
Wales	322	2.96 (2.31, 3.78)
**Wheezing**		
England	54	1.77 (0.98, 3.22)
Scotland	99	1.86 (1.18, 2.93)
Wales	88	2.31 (1.43, 3.74)
Pain		
**Chest pain**		
England	110	1.82 (1.19, 2.80)
Scotland	155	1.79 (1.25, 2.56)
Wales	155	2.45 (1.71, 3.51)
**Neuropathic pain**		
England	73	1.36 (0.78, 2.37)
Scotland	103	1.30 (0.81, 2.09)
Wales	109	1.55 (1.01, 2.39)
**Pain**		
England	936	1.24 (1.06, 1.45)
Scotland	1301	1.25 (1.10, 1.43)
Wales	1202	1.31 (1.14, 1.50)
Circulation		
**Limb swelling**		
England	76	2.69 (1.63, 4.43)
Scotland	101	1.47 (0.93, 2.33)
Wales	103	1.42 (0.90, 2.24)
**Palpitations / tachycardia**		
England	46	1.72 (0.88, 3.35)
Scotland	78	2.28 (1.39, 3.76)
Wales	66	3.26 (1.88, 5.66)
**Presyncope / dizziness**		
England	78	1.49 (0.89, 2.50)
Scotland	109	1.06 (0.67, 1.67)
Wales	111	1.35 (0.87, 2.10)
Fatigue		
**Fatigue / asthenia**		
England	135	3.17 (2.19, 4.58)
Scotland	246	2.91 (2.19, 3.87)
Wales	225	3.41 (2.53, 4.60)
Ear, nose and throat		
**Cough**		
England	159	1.50 (1.05, 2.13)
Scotland	242	2.47 (1.86, 3.28)
Wales	261	1.84 (1.40, 2.41)
**Ear pain**		
England	30	1.08 (0.40, 2.96)
Scotland	46	0.97 (0.47, 2.01)
Wales	58	1.08 (0.57, 2.06)
**Nasal congestion / sneezing**		
England	28	0.85 (0.33, 2.20)
Scotland	50	2.06 (1.11, 3.83)
Wales	56	0.72 (0.36, 1.43)
**Phlegm**		
England	29	1.86 (0.82, 4.19)
Scotland	61	3.09 (1.74, 5.47)
Wales	48	1.77 (0.94, 3.34)
**Sore throat**		
England	36	1.15 (0.51, 2.59)
Scotland	60	1.80 (1.02, 3.18)
Wales	69	1.61 (0.93, 2.80)
Stomach and digestion		
**Abdominal pain**		
England	140	1.41 (0.95, 2.08)
Scotland	205	1.31 (0.95, 1.82)
Wales	207	1.43 (1.04, 1.96)

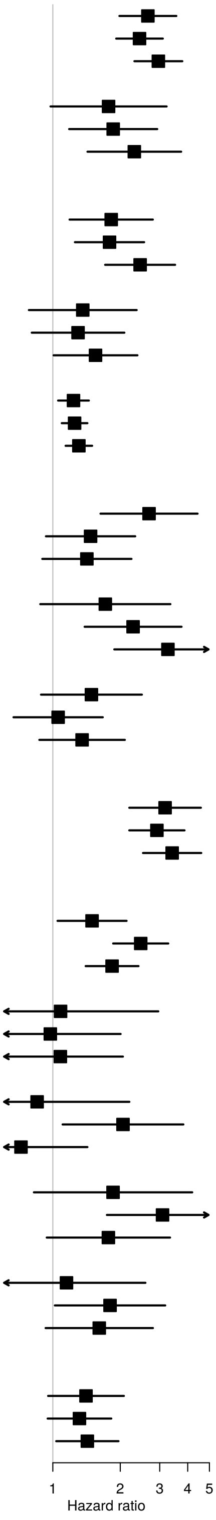

Symptom by domain	N events	Hazard ratio for symptom in confirmed COVID-19 versus unexposed
**Constipation**		
England	94	1.27 (0.79, 2.04)
Scotland	163	1.33 (0.93, 1.90)
Wales	134	1.10 (0.73, 1.64)
**Diarrhoea**		
England	78	1.79 (1.08, 2.96)
Scotland	137	1.32 (0.89, 1.95)
Wales	114	1.39 (0.89, 2.17)
**Gastric reflux**		
England	43	1.31 (0.64, 2.67)
Scotland	76	1.06 (0.63, 1.77)
Wales	81	1.93 (1.17, 3.19)
**Nausea / vomiting**		
England	155	1.47 (1.02, 2.13)
Scotland	231	1.76 (1.31, 2.37)
Wales	202	1.77 (1.29, 2.42)
**Weight loss**		
England	99	1.35 (0.85, 2.13)
Scotland	82	1.73 (1.06, 2.84)
Wales	105	1.28 (0.81, 2.01)
Muscles and joints		
**Joint pain**		
England	131	1.43 (0.96, 2.12)
Scotland	203	0.98 (0.70, 1.38)
Wales	154	0.88 (0.59, 1.31)
**Muscle pain**		
England	36	2.15 (1.04, 4.46)
Scotland	51	1.40 (0.71, 2.73)
Wales	64	1.85 (1.05, 3.26)
**Paraesthesia**		
England	83	2.57 (1.60, 4.12)
Scotland	125	0.92 (0.60, 1.43)
Wales	110	2.05 (1.34, 3.14)
Mental health		
**Anxiety / depression**		
England	243	1.68 (1.25, 2.26)
Scotland	374	1.39 (1.09, 1.76)
Wales	371	1.51 (1.19, 1.91)
Hair, skin and nails		
**Hives / itchy skin**		
England	44	1.37 (0.68, 2.75)
Scotland	84	1.19 (0.71, 2.00)
Wales	54	1.20 (0.61, 2.38)
**Purpura / rash**		
England	267	1.30 (0.98, 1.75)
Scotland	282	1.16 (0.87, 1.55)
Wales	292	1.16 (0.87, 1.54)
Other symptoms		
**Allergies / angioedema**		
England	46	1.28 (0.65, 2.52)
Scotland	77	1.31 (0.78, 2.22)
Wales	65	1.08 (0.59, 1.96)
**Chills and fever**		
England	93	0.94 (0.57, 1.54)
Scotland	98	1.88 (1.20, 2.94)
Wales	88	1.43 (0.89, 2.28)
**Headache**		
England	99	1.60 (1.02, 2.52)
Scotland	148	2.09 (1.44, 3.05)
Wales	152	1.44 (0.97, 2.13)
**Polyuria**		
England	50	0.84 (0.42, 1.70)
Scotland	68	0.88 (0.48, 1.61)
Wales	61	0.99 (0.54, 1.80)

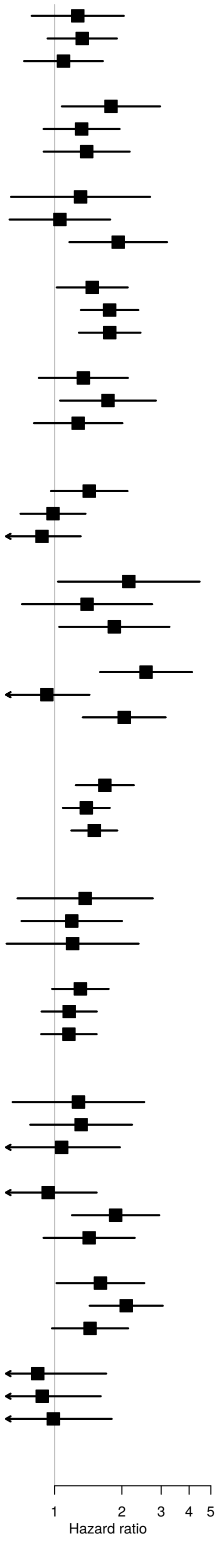

Supplement: S6 Fig — Association of symptoms with previous infection after 12 weeks, by UK nation. Hazard ratios were adjusted for age, sex, age/sex interaction, number of consultations in the year before the index date, number of symptom days 1–3 months before the index date, recording of the specific symptom 1–3 months before the index date, ethnicity, smoking, body mass index and a generated propensity score for acquiring COVID-19 infection, and stratified by general practice. (PDF) [file pone.0290583.s013.pdf]
