# Supplementary material for: Long Covid symptoms and diagnosis in primary care: A cohort study using structured and unstructured data in The Health Improvement Network primary care database
Source: PLoS One. 2023 Sep 26;18(9):e0290583. doi: 10.1371/journal.pone.0290583 (PMC10521988; doi:10.1371/journal.pone.0290583)

**Supplementary Figure S7: Elbow plot for latent class analysis**

Elbow plot from latent class analysis for symptoms occurring within 3 months of a WHO Long Covid symptom, among patients at least 12 weeks after a COVID infection. The inflexion point at 2 classes shows that additional classes do not improve the fit of the model. AIC, Akaike Information Criterion; BIC, Bayesian Information Criterion; SABIC, sample size adjusted Bayesian Information Criterion; CAIC, Corrected Akaike Information Criterion.

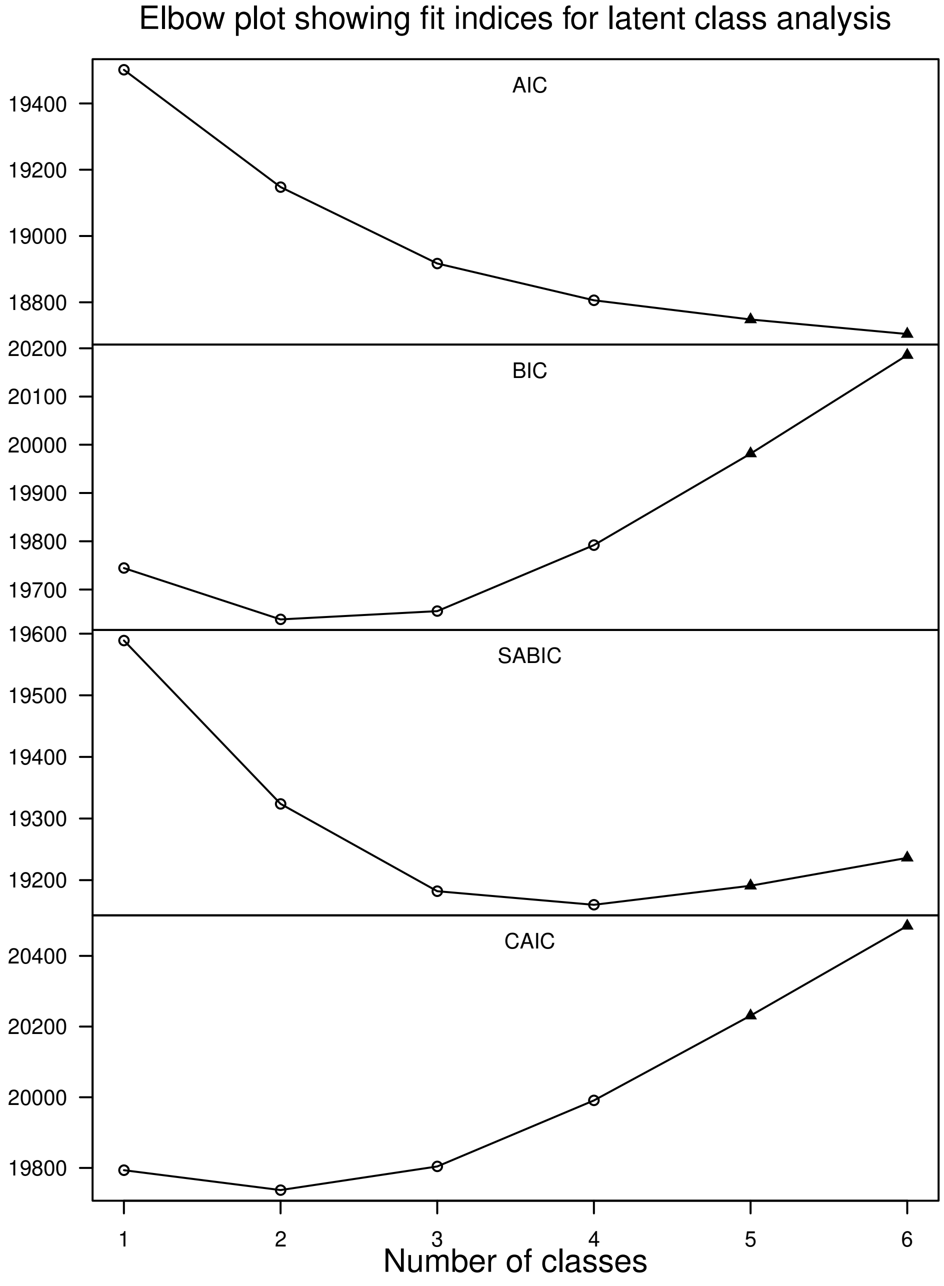

Supplement: S7 Fig — Elbow plot from latent class analysis for symptoms occurring within 3 months of a WHO Long Covid symptom, among patients at least 12 weeks after a COVID infection. The inflexion point at 2 classes shows that additional classes do not improve the fit of the model. AIC, Akaike Information Criterion; BIC, Bayesian Information Criterion; SABIC, sample size adjusted Bayesian Information Criterion; CAIC, Corrected Akaike Information Criterion. (PDF) [file pone.0290583.s014.pdf]
