# Supplementary material for: Long Covid symptoms and diagnosis in primary care: A cohort study using structured and unstructured data in The Health Improvement Network primary care database
Source: PLoS One. 2023 Sep 26;18(9):e0290583. doi: 10.1371/journal.pone.0290583 (PMC10521988; doi:10.1371/journal.pone.0290583)
Supplement: Attachment — Submitted filename: Response to reviewers.pdf [file pone.0290583.s015.pdf]

Reviewer Notes:

Reviewer #1: Alex McConnachie, Statistical Review

The paper by Shah et al reports on an analysis of data from THIN, using Read coding and NLP to identify symptoms reported in relation to Long COVID. This review looks at the statistical aspects of the paper.

Generally, these are very good. My comments are fairly minor.

The authors derive a propensity score for getting COVID, and use this to weight their analysis comparing those with COVID and those without. Was a single propensity score used, or were different ones derived for looking at confirmed COVID, or suspected COVID? A little more detail could be given about the weighting. Currently the paper says that analyses were "weighted by inverse probability", but the inverse probability of what, exactly? Normally, in an IPTW analysis, you weight the treated group by the inverse probability of being treated, and the untreated group by the inverse probability of being untreated. Was the same thing done here (replacing "treated" with "exposed")? I believe that approach gives an estimate of the average effect of the exposure if everyone were exposed, compared to if everyone were unexposed. Is that the right thing to do here? Are we more interested in the effect of exposure in those who are exposed? Should an "average treatment in the treated" -type of estimate be used? If so, then the weights used are different. As I say, more detail is required.

RESPONSE: Thank you for these comments. We generated 3 separate propensity scores for each comparison (confirmed COVID-19 versus unexposed, suspected COVID-19 versus unexposed, viral or respiratory illness versus unexposed). We have described the propensity score generation in detail in the Supplementary Methods.

In our original analysis we used inverse probability of treatment weighting (considering COVID-19 exposure as 'treatment', which would provide an average treatment effect estimate). However, we have now changed our method to include the propensity score as a covariate in a multivariable Cox model, which is recommended as an alternative method to IPTW (Elze et al. J Am Coll Cardiol. 2017;69(3):345-357. doi: 10.1016/j.jacc.2016.10.060). The reason for this change was that some estimates were unstable because of large weights caused by observations with extreme values of propensity scores. The change in method resulted in slight changes to some of the hazard ratios but no changes in direction of association or statistical significance.

New text (Methods: Comparison of symptoms in patients with and without COVID-19, paragraph 1): "Hazard ratios were adjusted for age, sex, age/sex interaction, number of consultations in the year before the index date, number of days on which any symptom was recorded 1-3 months before the index date, recording of the specific symptom 1-3 months before the index date, ethnicity, smoking, body mass index and a generated propensity score for acquiring COVID-19 infection which incorporates prior diagnoses according to the SNOMED CT hierarchy (see Supplementary Methods)."

Other than that, my comments are trivial.

I tend to report estimates in relation to age for a ten-year increase, to avoid confidence intervals that are very narrow.

RESPONSE: We have amended the estimates in relation to age so that they relate to 10 years older.

One of the limitations is given that the use of free text is subject to error, but decides that any misclassification is unlikely to have affected the hazard ratios. Is this true? Intuitively, one would think that any random measurement error would likely bias the results towards null.

RESPONSE: Thank you for pointing this out. We misrepresented this in the original text; we meant to say that misclassification would be unlikely to bias the findings towards demonstrating an effect when none is present. We have modified the text in Discussion: Limitations, paragraph 3 to "Therefore there may be some bias of hazard ratios towards the null but there is unlikely to be any significant over-estimation of hazard ratios due to differential misclassification."

Throughout the paper, the language often slips into talk of cases and controls, though this is not a case control study. I think "exposed" and "unexposed" would be more correct.

RESPONSE: Thank you for this comment, however as there are different types of 'exposure' in this study (e.g. confirmed or suspected COVID-19 infection) we have used the more descriptive wording 'confirmed / suspected COVID-19 case' and 'unexposed control' wherever possible.

Reviewer #2: This manuscript leverages Natural Language Processing from EHR records to better understand the symptoms that patients are experiencing post-COVID, and how these relate to a Long-COVID diagnosis. The adult cohort examined is from England, Scotland, and Wales and is approximately 11K patients compared to controls. Limitations of the study include having a relatively small cohort size, a lack of evaluation in different variant periods as well as different epochs post-acute COVID. The classification of propensity for long covid may need to take additional considerations into account, such as healthcare utilization and examination of the ICD U09.9 heterogeneity/bias, as well as considerations for defining long covid using only one or more symptom. Overall, the main theme of the manuscript seems to be to determine whether or not NLP can be used to extract key long covid symptoms from clinical notes. The findings that patients were more likely to have symptoms of Long Covid or receive a Long Covid diagnosis if they were older (although not necessarily advanced age), female, or hospitalized during COVID-19 is largely consistent with other studies. The manuscript might be better positioned within a clinical informatics journal rather than PloS Medicine for this reason. Specific comments follow:

RESPONSE: Thank you for these comments. As the study has both clinical and informatics aspects we have now transferred it to PLOS ONE, which is a cross disciplinary journal.

The manuscript background begins with reference to patient reported outcomes, which are a component of the WHO definition of Long-Covid. However, these are not utilized within the evaluation provided other than in the context of clinical notes. This seems somewhat of a mismatch in terms of background justification for the study? (it may well be that in the UK that patient reported outcomes are more commonly included in the free text notes than in other parts of the world). I do concur, however, that the field should provision "the accurate recording of symptom data, preferably in a structured way, in order to record and track a patient's disease over time and to facilitate research."

RESPONSE: Thank you for this comment. The motivation is to study Long Covid within the UK general practice setting, and in particular patient symptoms that are elicited and recorded by the GP. We have added more detail to background to clarify the contribution of this study. The study is not designed to allocate Long COVID diagnoses exactly according to the WHO definition because the complete information to do so is not in the patient record. We have changed the description 'Long Covid defined by WHO symptoms' to 'Presence of WHO Long Covid symptom' in Table 2 and Results: Clustering and risk factors for Long Covid.

For the NLP pipeline, it is not described whether synonyms of the terms were used, how context was determined (such as family history or past patient history or negation), and whether the sentence fragments were examined for co-occurrence or other factors that would be able to provision some sort of confidence score. The list of symptom strings in supplemental table 1 is somewhat weak from a computability point of view (many of these terms from standard terminologies have synonyms that could have been incorporated). No interannotator consistency measures appear to have been determined for the NLP manual review. This would be best practices in many contexts.

RESPONSE: We have added additional text describing the NLP pipeline in more detail in the supplementary material. We did include interannotator agreement in the supplementary text but we have now also mentioned this in the main text, at the end of the second paragraph of the Results section: "Inter-rater reliability of the manual annotators was good for symptoms (unweighted kappa 0.75, 95% CI 0.66, 0.83) but moderate overall (weighted kappa 0.54, 95% CI 0.48, 0.61)."

The Freetext Matching Algorithm (FMA) is not very recent or well known (not necessarily a problem); I am therefore having a hard time assessing its NLP parameters, such as whether it can leverage synonyms, stem matching, fragmented stems, or relationships between terms to improve accuracy. These would be more commonplace in today's NLP algorithms.

RESPONSE: The Freetext Matching Algorithm is described in detail in the paper Shah et al. BMC Med Inform Decis Mak 12, 88 (2012). https://doi.org/10.1186/1472-6947-12-88. The algorithm itself is available as open source software from https://github.com/anoopshah/freetext-matching-algorithm. FMA uses synonyms of concepts and a manually created synonym table of common clinical terms. It does not utilise stem matching, word embeddings or relationships between terms, but for this project it was chosen for ease of implementation and the fact that it had been tested on UK general practice records. We have included additional information about the algorithm in the supplementary material:

"FMA is a rule-based named entity extraction and linking algorithm. The initial text preprocessing stage consists of conversion to lower case, stopword removal, identification of dates, durations and numbers using regular expressions, and spelling correction (attempting single letter insertion or substitution, except the first letter of the word). The algorithm then identifies context (e.g. negation, uncertainty and experiencer) by matching patterns against a manually curated set of expressions.

The next step is to match sequences of up to five words to Read terms. If the text phrase does not match a Read term exactly, parts of the phrase are substituted by alternative words and phrases using a manually curated synonym table. A similarity score between text phrases and candidate Read terms is used to find the best match out of a set of potential matches. More details about FMA are included in the program documentation on GitHub (https://github.com/anoopshah/freetext-matching-algorithm)."

The examination of post-12 weeks for only a single potentially long-covid term does not necessarily indicate Long-Covid. Furthermore, it does not appear that patients with any of these symptoms prior to the COVID Dx were excluded as potentially being classified with long Covid? Further, it is not clear how a GP diagnosis of confirmed or suspected Long Covid is weighted by the proportion of each symptom recorded in the prior week.

RESPONSE: The purpose of this study was not to identify patients with Long Covid according to established diagnostic criteria, but to document symptom occurrence in a way that is agnostic to

any diagnosis label, and separately to investigate the diagnoses that GPs were currently recording for these patients.

We have added the following text to Methods: Clustering and risk factors for Long Covid:
"We did not attempt to assign a 'Long Covid diagnosis as this was not possible using the available data, instead we aimed to identify a patient population with symptoms that may be consistent with Long Covid for the purpose of the clustering analysis, similar to the approach used by Subramanian et al"

The objectives of the study were to describe the long term profile of symptoms as recorded in general practice for patients with COVID-19, however this really was not accomplished in terms of longer term trajectory. I would recommend evaluation at later time points as well or otherwise breaking up the phases further and comparing them.

RESPONSE: We fully agree with the reviewer's comments about following up patients for a longer period of time. Unfortunately this was not possible given the time limited permissions under which access to free text from primary care records was available, and we have stated that this is a limitation of this study.

An additional goal of the study was to identify symptoms associated with a GP diagnosis of 'Long Covid', and to explore clustering of Long Covid symptoms and risk factors for Long Covid. Again, given the lack of longitudinal evaluation/partitions, it is challenging to know how patients might cluster.

RESPONSE: The clustering analysis was exploratory in nature and we agree that longer term follow up and more detailed phenotyping would be ideal for this kind of study. We have changed the last sentence of the introduction to state that the clustering analysis was exploratory: "We also conducted an exploratory analysis of clustering of Long Covid symptoms and risk factors for Long Covid."

The paper doesn't cite some major relevant manuscripts that describe features of long Covid (both clinical and patient reported), use of the long covid ICD code, or clustering. I have included a few of those manuscripts here:

RESPONSE: Thank you for mentioning these recent manuscripts; we have now cited them.

Long covid phenotypes:
https://www.thelancet.com/journals/ebiom/article/PIIS2352-3964(21)00516-8/fulltext
https://www.thelancet.com/journals/eclinm/article/PIIS2589-5370(21)00299-6/fulltext

Clustering:
https://www.thelancet.com/journals/ebiom/article/PIIS2352-3964(22)00595-3/fulltext
https://pubmed.ncbi.nlm.nih.gov/35265728/
https://www.nature.com/articles/s41591-022-02116-3

Use of the U09.9 code:
https://bmcmedicine.biomedcentral.com/articles/10.1186/s12916-023-02737-6

The propensity score methodology seems underspecified. Specifically, How was SNOMED CT used to simplify the model specification? If anything given the specific list-focus of this work, it is not clear how SNOMED CT with its multiple classification would simplify things and no indication of how the predictors and/or their roll-up were specified (other than being in the 'Disorder'

hierarchy). Other factors such as age, sex, ethnicity, smoking, deprivation and body mass index seem appropriately included.

RESPONSE: We have described the propensity score method in more detail in supplementary material. As described in the response to reviewer 1, we have changed the analysis method to incorporate the propensity score as an additional covariate, rather than using weighting methods.

The authors state that numerous symptoms are reported to primary care at least 12 weeks after COVID-19, but only a subset are commonly associated with a GP diagnosis of Long Covid. This is to be expected given the lack of a gold standard clinical definition of Long-Covid and coding variability. It is not surprising that GPs may be using clinical codes but the rate of coding is low for long covid. It may be worthwhile to examine individual clinician diagnoses and their correlation/variability across clinicians with the reported structured and unstructured features.

RESPONSE: The number of patients with Long Covid diagnoses is small given the limitations of data availability for this study. However it would be very worthwhile to extend the timeline of the study and this is an argument for wider research and use of text. We have added as a research recommendation in the discussion: "It would also be of interest to investigate the process of recording of diagnoses among GPs using structured and unstructured data."

The authors found that socioeconomic deprivation was associated with a lower likelihood of a long Covid symptom or diagnosis. While the explanations are reasonable (inequality in access to care for example), the fact that these are not individually recorded socioeconomic factors at the patient level makes this conclusion challenging. Furthermore, the practice level socioeconomic information/index appears underspecified. Given the sensitivity of this conclusion, it would behoove the authors to apply more rigorous definitions and odds assessments.

RESPONSE: We agree that this is a preliminary finding and would ideally be further investigated using patient level data. This may be possible with future data linkages. We have added the following text to the discussion, in the limitations section: "… our data on social deprivation was at the practice rather than the patient level, so our estimates may have been affected by residual confounding by socio-economic factors, and we were unable to investigate whether patient-level deprivation affected the recording of symptoms and diagnoses by GPs."

Vaccination and date of initial vaccination availability as well as comparisons prior to covid-19 presence in the population could be used for comparisons or as controls in the latter case.

RESPONSE: We agree that vaccination would be useful to investigate, but very few patients had undergone vaccination in our cohort within the timescale of the data that was available.

Reviewer #3: This study uses natural language processing to extract symptom information from free text clinical notes to determine which symptoms are more commonly present among patients with long COVID. While the resulting list of symptoms appears accurate/plausible, in that it follows the trend of various long COVID phenotyping efforts, I do not feel that this work adds much in the way of novelty to the existing body of work in this space. I disagree with the authors' assertion that existing studies have small sample sizes and have "little information on which symptoms patients attend with." Links to example papers follow:

RESPONSE: Thank you for mentioning these additional papers, which we now cite in our manuscript.

Deer et al, 2021: https://pubmed.ncbi.nlm.nih.gov/34839263/

Al-Aly et al, 2021: https://pubmed.ncbi.nlm.nih.gov/33887749/
Estiri et al, 2021: https://pubmed.ncbi.nlm.nih.gov/34565368/
Pfaff et al, 2022: https://www.thelancet.com/journals/landig/article/PIIS2589-7500(22)00048-6/fulltext
Zhang et al, 2023: https://pubmed.ncbi.nlm.nih.gov/36456834/
Reese et al, 2023: https://pubmed.ncbi.nlm.nih.gov/36563487/
Pfaff et al, 2023: https://pubmed.ncbi.nlm.nih.gov/36093345/

The study period only goes through December 31, 2020. Much has changed in COVID research, treatment options, and patient presentations since that first year. COVID research moves very quickly--for a paper in 2023, I would like to see the date range expanded to account for more current data, at least through the end of 2021.

RESPONSE: We agree that it would be ideal to include more recent data within this analysis. Unfortunately the permissions under which data access was possible (particularly for the free text) were time limited, so it is not possible to retrieve additional source data. We propose that arrangements are put in place for broader access to free text for research within a safe governance framework.

Defining the "long COVID" category as the presence of even a single WHO symptom 12 weeks after COVID is extremely broad, and will result in many false positives. That list of symptoms includes items such as abdominal pain, joint pain, and anxiety which may be part of a long COVID diagnosis, but are not specific enough on an individual basis to serve as evidence of long COVID. Use of this definition will result in many false positives. Additionally, the authors appear to have used a different definition of long COVID for some analyses, namely whether "long COVID" is mentioned in patients' notes. Using multiple definitions for long COVID in the same paper is confusing.

RESPONSE: In this study we are not attempting to replicate the clinical application of WHO criteria to assign a diagnosis Long COVID; instead we are studying the presence of symptoms post COVID-19 in a way that is agnostic to any diagnostic label that might have been applied. We have amended the way that we refer to the study outcomes (e.g. a symptom from the WHO definition, or a GP diagnosis of Long Covid) in the legend of Table 2 and in Methods: Clustering and risk factors for Long Covid "We did not attempt to assign a 'Long Covid diagnosis as this was not possible using the available data, instead we aimed to identify a patient population with symptoms that may be consistent with Long Covid for the purpose of the clustering analysis, similar to the approach used by Subramanian et al."

I appreciate the use of NLP to augment the structured data, and agree with the assertion that many symptoms are only recorded in the free text. However, the authors state that "Associations observed with coded data were similar to those including free text, but the number of events was smaller so estimates were less precise." My interpretation of this is that the NLP was helpful, but not a game changer here, and that results would have been similar without it.

RESPONSE: Whilst in this study the results would have been similar with and without free text, the fact that only 20% of symptoms were recorded in the free text suggests that there is a risk of selection bias when using only structured data to estimate relative risk of symptom occurrence. A small difference in the probability of coding a symptom record between exposed and unexposed patients could potentially make a significant difference to outcomes from a structured data analysis. We assert that analysis of free text is important for validation of coded data where coding is far from complete.
