# Supplementary material for: Long Covid symptoms and diagnosis in primary care: A cohort study using structured and unstructured data in The Health Improvement Network primary care database
Source: PLoS One. 2023 Sep 26;18(9):e0290583. doi: 10.1371/journal.pone.0290583 (PMC10521988; doi:10.1371/journal.pone.0290583)
Supplement: Attachment — Submitted filename: response_to_reviewers.pdf [file pone.0290583.s016.pdf]

Response to reviewers: Long Covid symptoms and diagnosis in primary care: a cohort study using structured and unstructured data in The Health Improvement Network primary care database

August 2023

Reviewer #1: The value of this paper is the agnostic approach using open text analysis, since long COVID is still a condition which is not well understood. I have some questions regarding the design and the available data:
- The authors talk about cohorts and have 4 exposure groups. It is confusing when these groups are named cases and controls which suggest that they are describing the outcomes (the symptoms).

RESPONSE: We agree that the terminology is confusing. We have now removed the terms 'cases' and 'controls' and instead describe the exposure groups as four cohorts: 'Confirmed COVID-19', 'Suspected COVID-19', 'Viral or respiratory illness' and 'Unexposed'. We have entirely rewritten the 'Study population' section of the methods. The new text is:

> *We defined four fixed, non-overlapping patient cohorts among adult patients registered with a general practice contributing to THIN during the study period. Because of the need to use data from free text to help classify patients, and restrictions on access to the full THIN database, cohort selection was carried out in a stepwise manner (Figure 1). In the first step, exposed cohorts (confirmed or suspected COVID-19) were selected based on Read codes for suspected or confirmed COVID-19 (list of Read terms in Supplementary Table S1). Each patient's index date was defined as the date of their earliest record of COVID-19. Unexposed patients were then selected using a 1:1 matching algorithm to patients with confirmed or suspected COVID-19, in order to assign index dates to unexposed patients and ensure a similar distribution of key demographics between the cohorts. The matching variables were practice, age (+/- 3 years) and sex. We also defined a 'possibly exposed' cohort with Read terms for non-specific viral or respiratory illness (Supplementary Table S2). These were patients who had symptoms compatible with COVID-19 but no formal COVID-19 diagnosis. The index date for this cohort was defined as the earliest record of a non-specific viral or respiratory illness within the study period.*
>
> *We extracted free text notes for these initial cohorts and then refined the cohort selection based on COVID-19 diagnoses or test results extracted from the free text. We amended the index date if appropriate based on information in the free text; for example if a patient had a free text diagnosis of COVID-19 prior to their coded entry their index date was brought forward. If patients had a confirmed COVID-19 diagnosis they were included in the confirmed COVID-19 cohort regardless of other diagnoses recorded, and if patients had a suspected COVID-19 but not confirmed COVID-19 they were included in the suspected COVID-19 cohort whether or not they had a viral or respiratory illness recorded.*
>
> *We excluded patients registered for less than 1 year prior to the index date, or with less than 4 weeks ongoing registration after the index date, or if there were no consultations recorded at all (Figure 1).*

- The cohorts should be more clearly defined, are they fixed or dynamic cohorts?

RESPONSE: We have now stated in the text that these are fixed cohorts.

- Do all cohort members have prepandemic data on symptoms?

RESPONSE: Yes, all participants had to be registered with a participating practice for at least 1 year before the index date, and we expect that symptoms reported to the GP during that time would have been recorded.

- Are the patients their own controls when comparing symptoms before and after infections?

RESPONSE: No, we did not use the self controlled case series method (with each patient being their own control) because of temporal and seasonal changes which could influence symptom burden over time. This is why the primary comparison is between cohorts. However, prior symptom burden is used for adjustment in multivariable models.

- It seems that there was 1-to-1 matching between exposed (confusingly called cases) and unexposed patients for practice, age and sex. How is it then possible to estimate effects of sex and age, as is being done.

RESPONSE: Because of data restrictions, it was not possible to use the entire unexposed cohort and a selection procedure was therefore carried out for the unexposed cohort. Matching was used to generate an unexposed cohort with similar age and sex distribution to the exposed cohorts, and pseudo-diagnosis index dates that could be used for comparing symptoms over time. The matching was not used in analysis; regression models were unmatched and age and sex were included as covariates. This has now been explained more clearly in the methods, and the 'study population' section has been rewritten (see above).

- Are cohort participants clustered within practices? If so, is there any intra-practice correlation of symptoms?

RESPONSE: Our primary comparison was between exposed and unexposed patients. We assumed that recording practices (and therefore the baseline hazard of the symptom outcomes under investigation) may vary between practices, and wished to ensure that this did not affect the result. We therefore report analyses with the baseline hazard stratified by general practice.

- How is index date defined?

RESPONSE: The index date was the date of diagnosis of COVID-19 or a viral or respiratory illness for the exposed cohorts, and a pseudo-diagnosis date for unexposed patients. We have described the allocation of the index date in the 'Study population' section.

- In other studies of long COVID, symptoms that has to do with cognitive function ("brain fog" etc) are often described. Are these type of symptoms not picked up by the techniques used here?

RESPONSE: The natural language processing algorithm was designed to detect concepts in the text that currently exist in the Read terminology. The term 'brain fog' was not detected as it is not a Read term and had not been programmed into the model. Future NLP models can be trained to recognise this and other novel or non-standard symptom descriptions.

- It would be interesting if more data on the functional level of patients had been available, like ability to work or perform daily activities of living.

RESPONSE: We agree this would be ideal. We have added this sentence to the fourth limitation in the discussion:

> *Information on the functional level of patients, such as ability to work or perform daily activities of living, was not available.*

Reviewer #2: Shah et al describe an analysis of primary care EHR data to investigate symptoms associated with a diagnosis of long COVID. A particular strength of the study is the use of natural language processing to extract information from free text records that would otherwise be missed, and the work seems to provide a useful contribution to the literature (from my perspective as a statistician who has worked on COVID-19 studies). I have only relatively minor comments regarding the description of the study, along with some technical queries regarding the analysis.

As noted by previous reviewers, the dataset for analysis in this study is truncated at the end of December 2020. I do not think that this means that the analysis presented is no longer useful. However, given that the situation with long-COVID could now be quite different (given widespread vaccination and repeat infections with different variants etc.), I do feel that the timespan for the analysis needs to be specified in the Abstract and that the Introduction also needs to frame the analysis as effectively only considering the pre-vaccine period. These issues could also be explored in a little more depth in the Discussion.

RESPONSE: We have now mentioned the timespan of the study in the abstract and introduction. We have modified the Abstract methods to say:

> *We used primary care electronic health record data until the end of December 2020 ...*

RESPONSE: We have inserted this sentence into the last paragraph of the introduction:

> *Our study is based on data to the end of December 2020, i.e. the first wave of COVID-19 infections in a predominantly unvaccinated population.*

RESPONSE: In the first sentence of the discussion, we now mention that this is a pre-vaccination population:

> *By analysing primary care records including unstructured text from 60,800 patients across three UK nations during the early, pre-vaccination COVID-19 era, …*

RESPONSE: We have added the following sentence to the discussion section: symptoms following COVID-19 diagnosis:

> *The time period of our study (March to December 2020) meant that our study population was predominantly unvaccinated and the infections that occurred were with early variants of COVID-19. This should be taken into account when comparing our findings with those from more recent time periods.*

I have some queries regarding the matching process:
-Firstly, were controls required to be 'unexposed' for the entire analysis period? I think that in an ideal nested case-control study design, control status would only be defined at the index date of the case (with follow-up censored in the event of subsequent infection). I don't think that this should have had too large an impact for an analysis within this time period, but it would be a more important consideration for any analyses stretching into 2021 or 2022 given the very high cumulative incidence of SARS-CoV-2 at later time points.

RESPONSE: We have now mentioned the timespan of the study in the abstract and introduction. Unexposed patients were required to be unexposed for the entire duration of the analysis period.

RESPONSE: We agree that different analysis methods will need to be used for analyses in later years as the cumulative incidence of history of COVID-19 infection will be very high.

-How precisely was age matched?

RESPONSE: We aimed to match cohort distributions rather than carry out a matched analysis, but because of restrictions on data access it was most feasible to do this via preliminary patient level matching and then applying further eligibility criteria as per Figure 1. In the initial patient level matching, patients were matched on age (+/- 3 years), sex and practice. The original text also stated erroneously that patients were matched on ethnicity; this was originally intended in our analytic protocol, but was not carried out as there was a considerable amount of missing data and some ethic groups had small numbers. We have corrected the text.

-I found it quite confusing to work out how the authors ended up with their final cohort of cases and controls. This is not clear at all in the text, but Figure 1 is helpful. I am not sure why exclusions based on index date, registration date or available follow-up would be conducted after the initial matching process – were these initial oversights? Or did this relate to data access constraints? I can see why reclassification of cases based on free text analysis would happen after the initial matching given the analysis required to process this information, but this is not explained clearly in the text.

RESPONSE: Yes, this is correct, there were data access constraints which meant that it was not possible to obtain the free text until the cohorts had been provisionally defined based on coded data, and then additional exclusions were carried out subsequently when the data were accessible to researchers. This is now explained more clearly in the "Study population" section of the methods (see response to reviewer 1 above).

-If the analysis had retained 1:1 matching, then I would have suggested consideration of a frailty term in the analysis models for matched pairs, but I don't think that this is possible given the subsequent deviation from 1:1 matching. There is a related issue that the baseline hazard functions from index dates of controls don't really have a clear interpretation given the deviation from 1:1 matching to cases, but the overall balance in calendar period of follow-up for cases and controls should be largely retained despite the reclassification of some cases and controls.

RESPONSE: We apologise for the confusion in the descriptions. The cohorts are not considered to be matched for analysis. The unexposed cohort is effectively a stratified random sample intended to have a similar distribution of demographic characteristics to the exposed cohort.

Further minor comments:
-It is stated that "We stratified hazard by general practice…", I think that it would be correct and slightly clear to state "We stratified baseline hazard functions by general practice…".

RESPONSE: Thank you for this comment, we have modified the text and the figure captions.

-"No patients had a coded diagnosis of post COVID condition (Long Covid)…", I understand that the relevant ICD-10 code was not created until after the analysis period considered. This should perhaps be mentioned.

RESPONSE: Thank you for this comment. We have expanded on the delays in availability of terminology concepts in the discussion:

> *In our study we found that none of the Long Covid diagnoses were recorded using diagnosis codes. coded using Read termsThis was because the relevant SNOMED CT concepts (and linked Read terms used within GP systems) were not available for most of*

the study period. The UK SNOMED CT concept "Post COVID-19 syndrome" was released at the end of November 2020, and the International SNOMED CT concept "Chronic post-COVID-19 syndrome" was released at the end of January 2021. The ICD-10 code U09.9 "Post COVID-19 condition" was released in September 2020 [29]. Terminology updates in other coding systems were even more delayed; the US ICD-10-CM code for Long Covid was not available until October 2021 [9]. This shows the need for terminology systems to be updated in a timely manner to enable emerging conditions to be recorded faithfully.
